# Global spatially explicit yield gap time trends reveal regions at risk of future crop yield stagnation

**DOI:** 10.1038/s43016-023-00913-8

**Published:** 2024-01-26

**Authors:** James S. Gerber, Deepak K. Ray, David Makowski, Ethan E. Butler, Nathaniel D. Mueller, Paul C. West, Justin A. Johnson, Stephen Polasky, Leah H. Samberg, Stefan Siebert, Lindsey Sloat

**Affiliations:** 1 Institute on the Environment, University of Minnesota, St Paul, MN USA; 2 Project Drawdown, https://www.drawdown.org; 3 Université Paris-Saclay, INRAE, AgroParisTech, Palaiseau, France; 4 Department of Forest Resources, University of Minnesota, St Paul, MN USA; 5 Department of Ecosystem Science and Sustainability, Department of Soil and Crop Sciences, Colorado State University, Fort Collins, CO USA; 6 Department of Applied Economics, University of Minnesota, St Paul, MN USA; 7 Rainforest Alliance,; 8 Department of Crop Sciences, University of Göttingen, Göttingen, Germany; 9 World Resources Institute, Washington DC, USA

**Keywords:** Environmental impact, Agriculture, Sustainability, Socioeconomic scenarios

## Abstract

Yield gaps, here defined as the difference between actual and attainable yields, provide a framework for assessing opportunities to increase agricultural productivity. Previous global assessments, centred on a single year, were unable to identify temporal variation. Here we provide a spatially and temporally comprehensive analysis of yield gaps for ten major crops from 1975 to 2010. Yield gaps have widened steadily over most areas for the eight annual crops and remained static for sugar cane and oil palm. We developed a three-category typology to differentiate regions of ‘steady growth’ in actual and attainable yields, ‘stalled floor’ where yield is stagnated and ‘ceiling pressure’ where yield gaps are closing. Over 60% of maize area is experiencing ‘steady growth’, in contrast to ∼12% for rice. Rice and wheat have 84% and 56% of area, respectively, experiencing ‘ceiling pressure’. We show that ‘ceiling pressure’ correlates with subsequent yield stagnation, signalling risks for multiple countries currently realizing gains from yield growth.

## Abstract (web summary)

The patterns of how yield gaps change can suggest likely future outcomes for crop growth. This study conducts a spatial and temporal analysis of yield gaps for ten major crops from 1975 to 2010 and identifies regions where crops are experiencing ‘ceiling pressure’, signalling opportunities to improve future food security.

## Main

The green revolution coincided with a doubling of global crop production from the late 1960s to 2000 (ref. ^1^), alleviating fears of a Malthusian crisis. However, these production increases have come at a substantial environmental cost, and steadily increasing food demand is placing additional pressure on natural resources^2,3^. Closing yield gaps is widely cited as a pathway for increasing production while minimizing environmental impacts^1,2,4–9^ and is directly related to several of the United Nations Sustainable Development Goals^10^, including no poverty, zero hunger, decent work and economic growth, climate action and life on land.

Considerable efforts have been devoted to calculating yield gaps at local to global scales, using various complementary methodologies, all of which compare current yields (measured or modelled) to a yield ceiling^11^. Conceptualizations of yield ceiling range from agronomic potential^12,13^ to best-in-class regional yield^6,14–16^. We use as yield ceiling an ‘attainable yield’ defined as the 95th percentile observed regional yield, intending to estimate the highest yield attained somewhere in the world in each set of biophysical conditions. This definition of attainable yield follows that of Evans and Fischer^17^ and is effectively the same as the ‘feasible yield’ defined by van Dijk et al.^18^ or the ‘plateau’ in exploitable yield as articulated by van Ittersum et al.^8^, which remains 15–25% below^17^ agronomic potential^8,18–21^.

Most yield gap studies to date have been snapshots in time and lead to limited policy recommendations. Van Oort et al.^22^ classify yield gap studies into ‘narrow scope high detail’ relevant to analysing specific interventions but with limited range of applicability, and ‘broad scope low detail’ that can address questions about the envelope of sustainable production possibilities or indicate locations to target agronomic intervention^22^. Many authors have evaluated the envelope of sustainable production based on static yield gaps^5,6,22,23^, but there have been limited attempts to derive policy recommendations from these studies^22^. Van Oort et al.^22^ identify five categories based on a combination of economic, climatic and humanitarian considerations, and recommend specific policy interventions ranging from increasing resource use efficiency to agricultural research and development^22^. Fischer and Connor assess yield gaps and divide the world into two typologies: low input–large yield gap, indicating a need for improving farmers’ access to tools for management, and intensified small yield gap, indicating a need for increases in potential yields^24^.

A few previous studies have considered yield gap trends through time. A monograph by Fischer et al. surveys trends in yields and yield ceilings based on case studies covering multiple crops and regions^23^. However, this analysis does not have consistent global coverage. Fischer et al.^23^ reach policy conclusions consistent with those of Fischer and Connor^24^. Hatfield and Beres derive yield gaps for wheat for ten countries based on a quantile regression analysis of national time series of yields supplemented by state- and county-level data^16^. Such studies are valuable contributions and can provide a greater understanding of both production possibilities and specific policy prescription than snapshot-in-time studies. We aim here to provide a study that is more comprehensive in number of crops, spatial resolution of data and global coverage and draw policy-relevant conclusions on trends in global production potential and indications of desirable interventions for assuring food security.

We calculate spatially explicit global time trends in attainable yields and yield gaps from 1975 to 2010 for ten crops comprising 83% of global calories (maize, wheat, rice, oil palm, soybean, barley, sugar cane, sorghum, rapeseed and cassava). We use a quantile regression model with year-specific coefficients to calculate the area-weighted 95th percentile yields for each year across the world given local climate, soil characteristics and irrigation management. These 95th percentile yields are designed to quantify the best yields in each set of biophysical conditions, denoted the ‘attainable yield’. The present method extends climate analogue approaches^6,14^ with inclusion of a broader set of biophysical variables and methods that result in continuous yield surfaces, few parameters relative to process-based models and calculation of confidence intervals. Our analyses are based on a high-resolution historical crop dataset derived from census and survey information across ∼20,000 political units. We use a static climatology that leads to more accurate models of yield gap trends than those based on yearly data. To facilitate comparison between crops and time periods, we quantify growth as percentage of linear change relative to 2000 yield values, where possible. We chose 2000 as a well-studied baseline^2,6^ considered the end of a phase of the green revolution^1,25^. We report results for the globe and eight geographical regions. Detailed results are presented in Supplementary Information for countries whose production exceeds 1% of 2000 production.

## Results

### Yield gaps are dynamic

Consistent with a history of growth in actual yields^12,26,27^, attainable yields have increased from 1975 to 2010 over a majority of areas (Table 1 and Supplementary Fig. 1). Attainable yields increased over more than 94% of the 2000 harvested area between 1975 and 2010 for six crops (maize, rapeseed, rice, sorghum, soybean and wheat). Cassava and barley had attainable yield growth over 85% and 69% of harvested area. Two perennial crops, sugar cane and oil palm, experienced growth in attainable yield over 56% and 7%, respectively, of the year 2000 harvested area (Table 1 and Supplementary Table 10). The extent of areas undergoing growth in attainable yield has changed dramatically across the first and last decades in the study. Barley, rapeseed, sorghum and wheat have seen an order of magnitude drop in area of attainable yield growth, while cassava went from 0 to 75%. Maize and soybean show an increase in attainable yield over 85% of area in all decades (Table 1 and Supplementary Table 1).

**Table 1:** Percentage of harvested area with growth in attainable yield and average annual percentage growth in attainable yields

	Percentage of harvested area with significant growth in attainable yield			Average annual percentage growth in attainable yield		
	Entire period	First and last decades		Entire period	First and last decades	
	1975–2010	1975–1985	2000–2010	1975–2010	1975–1985	1975–2010
**Barley**	69%	43%	1%	0.8%	1.3%	0.7%^a^
**Cassava**	85%	0%	75%	0.8%	0.1%^a^	3.4%
**Maize**	100%	88%	97%	1.4%	1.4%	1.5%
**Oil palm**	7%	0%	1%	1.3%	1.6%^a^	0.3%^a^
**Rapeseed**	99%	86%	1%	1.6%	2.2%	2.1%
**Rice**	100%	94%	65%	1.2%	1.8%	0.7%
**Sorghum**	94%	53%	0%	0.1%^a^	0.5%^a^	0.3%
**Soybean**	100%	89%	86%	1.1%	1.1%	1.0%
**Sugar cane**	56%	6%	0%	0.4%	0.6%^a^	−0.2%^a^
**Wheat**	98%	78%	4%	1.3%	1.8%	0.7%^a^

Areas for which attainable yields increase over the indicated period with 95% confidence intervals (first three numerical columns) and average annual per-year linear growth in globally averaged attainable yield (last three numerical columns). Statistical analysis was carried out independently for the full interval as well as the first and last decades. Calculations of area and attainable yield growth are relative to 2000 for the 1975–2010 and 2000–2010 intervals, and relative to 1975 for the 1975–1985 interval. Confidence intervals and regional results are shown in Supplementary Table 11. Results with fixed-area counterfactuals are shown in Supplementary Table 3.

^a^Not significant (95% confidence intervals encompass zero).

The rate of attainable yield growth shows variation across crops, regions and time periods with attainable yield growth at the global scale over 1975–2010 for all crops except sorghum (Table 1 and Supplementary Tables 3, 10, 11 and 15–24). Maize, rapeseed and soybean show >1% growth in attainable yield over the 1975–2010 period and in the latest decade. While wheat and rice also experienced >1% growth over the full interval, growth rates have fallen in the most recent decade. Cassava stands out for a large increase in attainable yield growth in the most recent decade (Table 1 and Supplementary Table 2).

Yield gaps have increased over areas ranging from 10% (oil palm) to 71% (maize) of the 2000 harvested area (Table 2 and Supplementary Figs. 2 and 3) between 1975 and 2010. Rice and wheat have substantially less area with growing yield gaps in 2000–2010 than in 1975–1985 (Table 2). By contrast, both maize and soybean have growing yield gaps in more than 37% of area for all periods studied (Table 2 and Supplementary Table 4).

**Table 2:** Percentage of harvested area with growth or a decrease in yield gaps

	Area with significant growth in yield gap			Area with significant decrease in yield gap		
	Entire period	First and last decades		Entire period	First and last decades	
	1975–2010	1975–1985	2000–2010	1975–2010	1975–1985	2000–2010
**Barley**	36%	21%	1%	4%	1%	1%
**Cassava**	48%	0%	46%	13%	15%	3%
**Maize**	71%	41%	55%	16%	14%	14%
**Oil palm**	10%	0%	0%	7%	0%	4%
**Rapeseed**	52%	29%	5%	5%	3%	1%
**Rice**	56%	58%	15%	24%	9%	24%
**Sorghum**	62%	28%	5%	7%	8%	2%
**Soybean**	61%	44%	37%	15%	10%	17%
**Sugar cane**	28%	6%	4%	22%	3%	13%
**Wheat**	62%	36%	5%	8%	1%	4%

Areas where yield gaps increase or decrease over the indicated period with 95% confidence intervals. Area calculation is relative to 2000 for the 1975–2010 and 2000–2010 intervals, and relative to 1975 for the 1975–1985 interval.

**Fig. 1:**
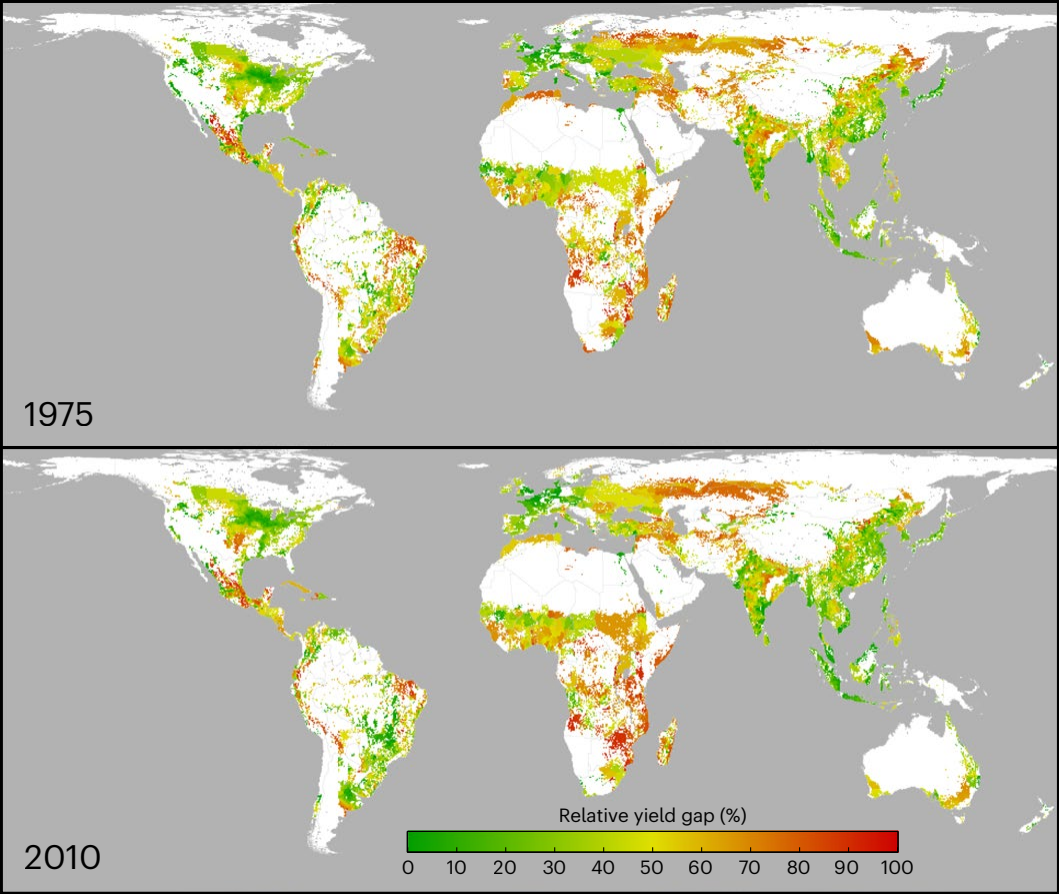
Average relative yield gaps for ten major crops in 1975 and 2010. Crops include barley, cassava, maize, oil palm, rapeseed, rice, sorghum, soybean, sugar cane and rice. Relative yield gap (shown as the percentage of the attainable yield achieved) in each grid cell is calculated as an area-weighted average across the crops and is shown on the top 98% of the growing area.

Globally averaged yield gaps have increased for barley, maize, rapeseed, rice, soybean and wheat from 1975 to 2010, with no significant change for cassava, oil palm, sorghum or sugar cane (Table 3 and Supplementary Fig. 2). Only maize, rapeseed and soybean have seen growing yield gaps in both the first and last decade of this interval, whereas rice and wheat went from growth in yield gaps of nearly 2% per year in 1975–1985 to no growth in the 2000–2010 interval (Table 3). Regionally, there is heterogeneity in yield gap change, with substantial areas showing significant decreases in yield gaps (Supplementary Table 12). Averaged relative yield gaps for 1975 and 2010 are shown in Fig. 1, revealing regions where yield gaps have closed (for example, eastern Asia, Brazil, Australia) and widened (for example, sub-Saharan Africa, eastern Russia.)

**Table 3:** Average annual percentage change in globally averaged yield gaps

	Average annual trend in yield gap		
	Entire period	First and last decades	
	1975–2010	1975–1985	2000–2010
**Barley**	0.9%	1.7%^a^	0.3%^a^
**Cassava**	0.6%^a^	−1.9%^a^	5.6%
**Maize**	1.2%	1.1%	1.6%
**Oil palm**	−0.4%^a^	−0.7%^a^	−2.6%^a^
**Rapeseed**	1.5%	2.1%	1.5%
**Rice**	0.8%	2.1%	−0.4%^a^
**Sorghum**	0.4%^a^	−0.2%^a^	0.8%^a^
**Soybean**	0.8%	1.1%	1.4%
**Sugar cane**	0.1%^a^	0.6%^a^	−1.3%^a^
**Wheat**	1.2%	1.9%	0.1%^a^

Reported change is per-year linear change relative to 2000 yield gaps for the 1975–2010 and 2000–2010 intervals, and relative to 1975 for the 1975–1985 interval. Confidence intervals and regional results are shown in Supplementary Table 12. Means are calculated after rejecting outliers (some realizations have yield gap ∼0, leading to infinite relative growth rates). Outliers are defined as points outside of the interval mean + 4s.d. Results with fixed-area counterfactuals are shown in Supplementary Table 3b.

^a^Not significant (95% confidence intervals encompass zero).

### Yield gap closure correlates with yield stagnation

Agricultural census units where the yield gap is closing have a greater likelihood of future yield stagnation than census units where the yield gap is static or widening. For all crops studied except oil palm, significant yield gap closure from 1986 to 2000 increases the likelihood of yield stagnation from 2000 to 2012 by factors ranging from 1.78 (sugar cane) to 3.75 (soybean) (Supplementary Table 5). Here we define yield stagnation following Grassini^28^ as a plateau after a period of growth. This increase in stagnation likelihood is not merely a ‘reversion to the mean’ effect associated with high yield growth rates: limiting analysis to regions in the top yield-growth quartile leads to qualitatively similar results (Supplementary Information).

**Fig. 2:**
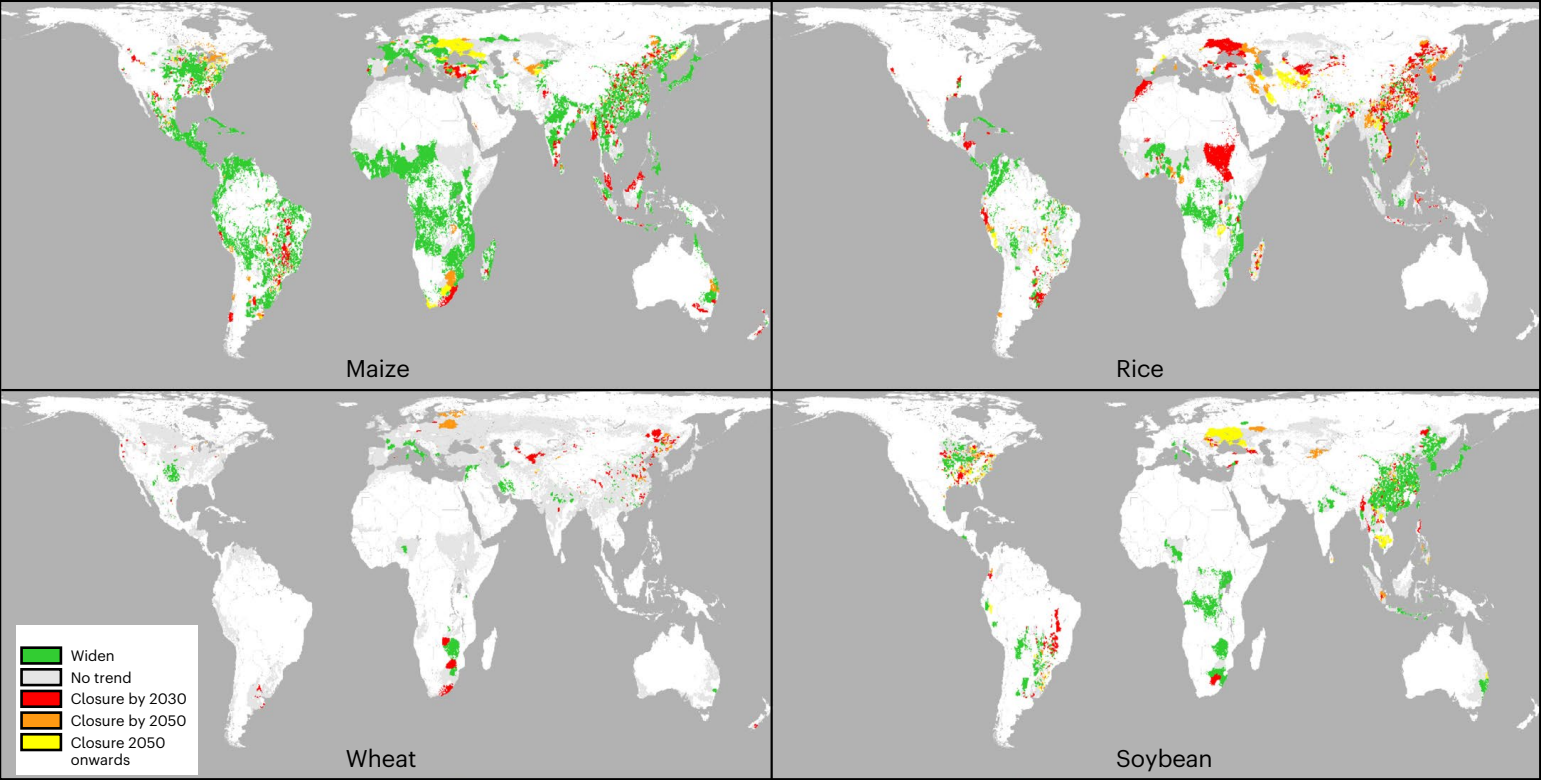
Time to closure of yield gaps based on linear extrapolation of trends from circa 2000 to circa 2010 for maize, rice, wheat and soybean. Yield gap closure time is defined as the crossing point of the linear trend of attainable yield and the linear trend of actual yield relative to 2010. ‘No trend’ indicates that no yield gap closure occurs within 95th percentile confidence intervals. ‘Widen’ indicates that yield ceiling and linear trend are significantly diverging (that is, the crossing point is before 2010). See Supplementary Fig. 3 for other crops.

We evaluate trends in yield gap over the period 1998–2012 to identify areas at risk of future yield stagnation. For rice, yield gaps have a significant rate of closure over 23% of area, representing 27% of 2000 rice production, and are on track to close by 2030 for 18% of 2010 area (Fig. 2 and Table 2). Soybean yield gaps are closing over 16% of area (Table 2). Yield gaps for maize, in contrast, are growing significantly over 56% of area and closing over 13% while only 3% of maize area is trending towards closure by 2030 (Fig. 2 and Table 2). Yield gaps are widening in more areas than they are closing for multiple crops (Table 2).

Rates of gap closure are particularly acute in some countries and regions. For example, 11%, 51% and 34% of rice production in Indonesia, Bangladesh and Vietnam (the world’s 3rd, 4th and 5th largest producers), respectively, are on a trajectory to close yield gaps by 2030 (Supplementary Information). In China, the world’s largest wheat producer, 11% of harvested area has yield gaps closing by 2030. In Thailand, 86% of sugar cane production trends to yield gap closure by 2030.

### Yield gap typologies reveal trends relevant to food security

**Fig. 3:**
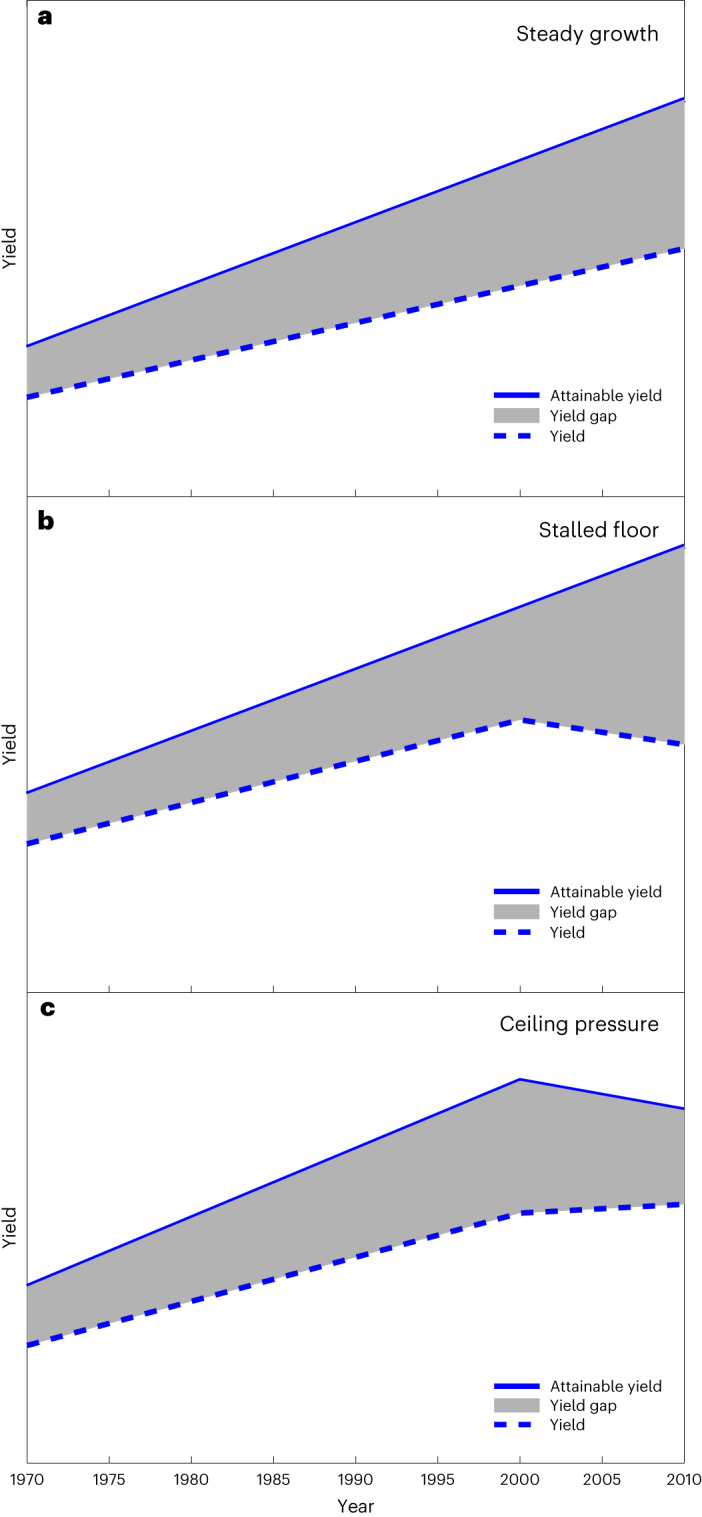
Typologies of yield gap closure. **a**, ‘Steady growth’ category, an archetype of which is the situation when attainable yield (‘ceiling’) and actual yield (‘floor’) benefit from agronomic investment in new technologies and increased uptake of management practices. **b**, ‘Stalled floor’ category; yield gap is increasing because ‘best in class’ management practices for maximizing yield are not widely adopted for reasons that could include economic barriers, selection of lower-yielding higher-quality cultivars or adoption of environmental policies. **c**, ‘Ceiling pressure’ category, in which small yield gaps indicate a need for breeding and ‘new agronomy’^24^ to improve yield ceilings. Some archetypal examples are shown in Supplementary Fig. 5.

Linear yield gap trends cannot by themselves differentiate underlying causes of change, for example, stagnation in yield versus growth in attainable yield. We introduce a three-element typology of yield gap trajectories (Fig. 3), including ‘steady growth’ in which yield gaps grow while attainable yield (‘ceiling’) and actual yield (‘floor’) increase, ‘stalled floor’ in which attainable yield grows but actual yield stagnates and ‘ceiling pressure’ in which yield gaps close and/or attainable yield stagnates.

**Fig. 4:**
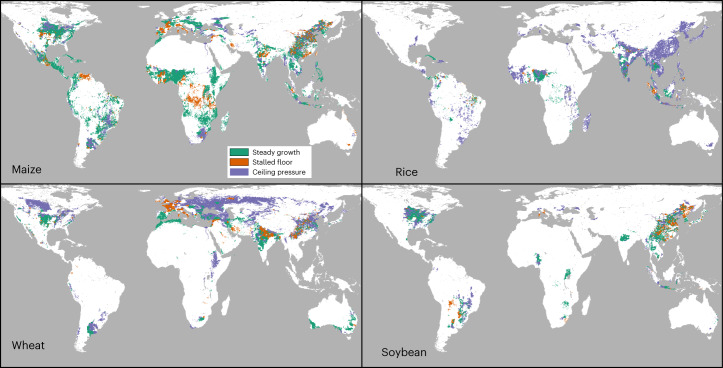
Maps of typologies of yield gap change for maize, rice, wheat and soybean. Typologies are as defined in the text and illustrated in Fig. 3. Maps for other crops are shown in Supplementary Fig. 4.

Yield gap trends are experiencing steady growth over half the harvested area for cassava (65.2%), maize (60.3%) and sorghum (51.2%). By contrast, rice has the lowest percentage of harvested area experiencing steady growth (12%) and 84% is experiencing ceiling pressure (Figs. 3 and 4, Table 4 and Supplementary Table 13). The crop with the second greatest area experiencing ceiling pressure is wheat at nearly 56%. All crops have some area with stalled floor, ranging from 3.9% (rice) to 18.7% (cassava.)

**Table 4:** Global allocation of circa 2000 harvested area into three typologies of yield gap evolution

	Steady growth	Stalled floor	Ceiling pressure
	Area	Area	Area
**Barley**	40.6%	6.4%	53%
**Cassava**	65.2%	18.7%	16.1%
**Maize**	60.3%	14.6%	25.1%
**Oil palm**	39.7%	10.7%	49.6%
**Rapeseed**	42.2%	11%	46.8%
**Rice**	12%	3.9%	84.2%
**Sorghum**	51.2%	9.1%	39.6%
**Soybean**	48%	11.2%	40.8%
**Sugar cane**	28.8%	18%	53.2%
**Wheat**	28.8%	15.5%	55.7%

## Discussion

These results inform a long-running debate on potential for future growth in yields. In general, we find that both actual yields and attainable yields have continually improved over many decades^8,12,26,27^. However, the nature of how they have grown—and the evolution of yield gaps—sheds light on contrasting claims regarding likely trajectories for future yield growth. One view is that historical growth in crop yields is due to ‘one-time innovations’^28^: a ceiling is being approached, and meeting demand beyond 2030 will require novel technological advances^12^. This could be the situation for rice: over 3 decades, the ratio of harvested area with yield gaps growing versus shrinking flipped from 6:1 to 1:2 (Supplementary Table 4) with 84% of rice area now experiencing ‘ceiling pressure’ (Supplementary Table 13). A contrasting view has been that economic incentives will lead to continuous future improvement^29^ and that increases in agricultural research and development (R&D) will lead to further increases in crop yield^30,31^. Maize trends are consistent with this view, with increases in production in areas with diverging yield gaps increasing over the decades (Supplementary Table 4) and 60% of maize area experiencing ‘steady growth’ (Supplementary Table 13). The present approach represents a possible method for relating yield outcomes to investment in agricultural R&D and extension that will be critical to achieving food security and climate goals^32^.

Regions experiencing ‘ceiling pressure’ have closing yield gaps and are at risk of a pronounced decrease in future yield growth (if not outright stagnation) in the absence of investment to raise attainable yields. While this result is intuitive, we have quantified the effect and shown that measures based on yield gap closure rates are more reliable predictors of future stagnation than measures based on local yield time series or size of yield gap. By calculating yield gap closure over an equivalent time interval, we identify risk of future stagnation for multiple regional crops, particularly rice in Asia, with Vietnam, Bangladesh and China at heightened risk with 75%, 59% and 34%, respectively, of production in areas where yields are approaching the ceiling (Supplementary Fig. 4 and Supplementary Information). Wheat yields in many European countries also have much harvested area undergoing ‘ceiling pressure’ (Supplementary Fig. 4 and Supplementary Information), consistent with the yield stagnation observed by other authors^33^. Policies to address ‘ceiling pressure’ include those that increase yield ceilings, notably investment in breeding technologies and improved agronomic practices^24,23^. Closing yield gaps in China have been noted along with calls for increased investment in management technology and advanced cultivars^34^.

The ‘stalled floor’ category is characterized by a significant decrease in growth rate of actual yield coupled with an increase in the yield gap. There can be multiple reasons for this, ranging from economic shocks to adoption of environmental policies to lack of investment in agronomics. Practices in western Europe intended to provide environmental benefits may lead to stalling yields because such practices (for example, reduction of fertilizer inputs, adoption of agro-ecological systems, banning of chemical pesticides) do not aim solely to maximize yields but rather to balance trade-offs with environmental impacts^35^. A related example is legislation in Italy limiting the uptake of genetically modified strains of maize that can resist pests has caused yields to decrease^36^ (Supplementary Fig. 5). Agronomic decisions can also lead to decreases in yield growth. As yield is reported in units of tonnes per cultivated hectare, this can be associated with increased productivity in conjunction with increasing multicropping, particularly prevalent for rice^37^, but can also come about because a crop is less favoured, such as sorghum in the United States being displaced by maize on the most productive farmland^23^.

Yield gaps evolve with change in either yield ceiling or floor, and ‘steady growth’ reflects ongoing increase in both, characterized by consistent growth in attainable yield, associated with investment in agricultural R&D, and growth in actual yield, associated with diffusion of improved management practices and results of R&D. An example of this category is sorghum in India (Supplementary Fig. 5), where a strong programme of private–public partnership led to the development of private plant breeding and uptake of improved hybrids^23^. Maize in many countries (Fig. 4) is undergoing this steady growth. In Mexico, this trend is evident (Supplementary Figs. 4 and 5) in spite of relatively large yield gaps associated with farmers growing lower-yielding white corn preferred by consumers^38^.

Yield gaps can vary with year-to-year on-field practices, but also with structural changes, such as lower-productivity land coming into cultivation. Yield ceilings for most crops have decreased compared with a fixed-area counterfactual, indicating that production has shifted to areas with lower yield ceilings (Supplementary Table 3) with oil palm, rapeseed and sugar cane as exceptions. Similarly, yield gaps for all crops except rapeseed have decreased compared with the fixed-area counterfactual suggesting that increases in cultivated area tend to occur in regions with higher yield attainment. This is consistent with Jevon’s paradox of intensification leading to increased landcover change.

Recent yield gap trends (circa 2000 to 2010) are significantly different for maize and soybean than for wheat and rice. Maize and soybean, which respectively deliver 24% and 52% of calories directly or indirectly as food^39^, have significantly increasing yield gaps. By contrast, wheat and rice, which deliver over 78% and 86%, respectively, of their calories as food^39^, have seen no significant change in yield gaps. This result is probably due to increased investment for maize and soybean, correlated with net production value increases over this time period of 289% and 292% compared with increases of 106% and 87% for wheat and rice, respectively^27^. Thus, ‘steady growth’, with its attendant potential for future yield improvement^12^, is occurring for crops that largely do not feed people (directly). An optimistic reading of this is that commensurate levels of investment are possible for crops that are more critical to food security, and a growing body of research suggests that R&D investment in crops critical to food security can improve yields^31,40,41^ and will be required to meet future food demand^12^. Recent research, however, shows that the temporal relationship between R&D expenditure and yield improvements exhibits longer lag times and more uncertainty than previously estimated, especially for developed countries^42^. Such lags increase the urgency of making R&D investments.

The quantile regression approach, based on a large census-based empirical yield dataset and global biophysical dataset, complements other possible methods for calculating yield gaps over time. Experimental approaches to calculating yield potential can be highly accurate for specific locations^23^, but difficult to generalize across space and time owing to cost and the difficulty of representing a larger region. Running process-based models at point locations to calculate potential yield can incorporate detailed information about soils, weather and management and be more representative of a larger region^8,43–46^. However, results from such models can depend on the details of how the models are parameterized^47^. Unlike experimental and process-based approaches, the global quantile regression approach used here is sensitive to the dynamic, real-world changes in cultivar and management as it is based on census and survey data at administrative units around the world. However, the present approach also has limitations owing to the scale and type of information that can be used in a global model. For example, we may overestimate yield gaps in Africa because of limited soil rooting depth^48^. Variables such as slope, soil organic carbon and pH are significant in explaining yield quantiles, but using these model terms to predict results of extensification requires detailed assumptions about the soil properties of land available for expansion. These results do not by themselves speak to the constituent components of yield gap, a topic that can be usefully addressed with process-based approaches^18^. The focus of our study is temporal trends in attainable yields—application of similar methods using cross-validation in physical space and climate space would result in attainable yield surfaces more appropriate for the assessment of production gaps. While there may be crop and climate combinations that do not experience advanced management leading to conservative predictions of attainable yield, the trends will still be valid. Global approaches can show sensitivity to choice of weather variables^45,49^ although we find our results to be robust with regard to selection of climatic datasets (Supplementary Information). The present method is explicit with regard to choice of weather variables and climate dependencies (Tables 5 and 6).

The present approach, with its global scope and basis on empirical yield data, emphasizes policy relevance over agronomic precision. Indeed, top-down frameworks such as the one used here can lead to instances at a local scale of predictions of yield ceiling below current production^46^. By using census-based yields for the empirical comparison, this method sidesteps the need to untangle exploitable and agronomic potential^8,11,19–21^. The global nature of the dataset provides a framework for sustainability studies. Moreover, while the approach is global, it has predictive power at the local scale as evidenced by the skill it shows to predict likelihood of yield stagnation. The inclusion of irrigated fraction is another important benefit, allowing for the exploration of scenarios with changes in irrigation; until now, this has been a notable drawback of analogue approaches^8^. This approach complements other global approaches such as the Global Agro-Ecological Zones^50^, finding similar quantitative results at a global scale for a different set of crops (Supplementary Fig. 6) while providing trends in attainable yield.

Recent climate change does not alter the conclusions of this analysis. Because our method fits model parameters anew every year, climate change impacts on yield will be mirrored in changes to attainable yield; thus, yield gap calculations are only indirectly sensitive to change in yield due to climate. However, future climate change could impact projections from this analysis owing to factors beyond the scope of this model such as yield losses due to increased pathogen risk^51^ or increased yields due to adaptations of cultivars and cropping calendars^52^. Despite this, a recent econometric analysis argued that historical adaptation to climate change is negligible^53^ and actual yield losses from increased pathogen risk are not well quantified and increased primarily in regions with yield gains from a changing climate^51^. Furthermore, future climate change will shift the likelihood of extreme conditions, which may also drive extreme yield losses^54^. While extreme climate change would lead to the functional form of the yield surfaces being poorly matched in later years, there are two reasons to discount this here. One is that impacts on yield due to climate change over the time period studied are typically smaller than growth due to evolution in cultivars and management^26,55–57^. A second reason is more concrete: we repeated analysis with yearly weather data instead of a fixed climatology and found that the latter led to lower temporal cross-validation errors (Supplementary Information). In short, while future climate change may move the global system into a climate space that is poorly captured by this model, the historical analysis presented here appears robust to climate change experienced to date.

Identifying yield gaps is not the same as prescribing how to close yield gaps. While this analysis looks to identify yield gaps and categorize regimes of yield gap evolution, it does not offer prescriptions regarding how those gaps should be addressed. The very concept of closing yield gaps is not value-neutral and can be problematic^58^. In addition to the difficulties of addressing the myriad socio-economic factors that keep farmers from improving production, small yield gaps imply reduced potential for future growth^20,58^. In some contexts, for example, in which smallholder subsistence agriculture is prevalent, significant investment in closure of yield gaps in primary crops may not be appropriate. This might be the case in which the investments in inputs required to increase yields lead to high levels of debt for farmers, especially where availability of inputs may fluctuate year to year, leaving farmers open to risk of over-investment in specific crops or crop varieties. Singular focus on yields of staple crops may also occur at the expense of diversified crop production, with negative consequences for local nutrition and food security. In the context of transnational land acquisitions, closing yield gaps can increase production while putting local food security at risk^59^. By contrast, Zhang et al.^60^ report that intense agricultural outreach to smallholder farmers in Quzhou, China, helped close yield gaps and increase farmer incomes. Yield gaps may also persist as reflections of consumer preferences (for example, white corn in Mexico^38^ or high-protein low-yield wheat^61^).

Since 2000, the growth rates in yield gap experienced by maize, soybean and rapeseed over the green revolution have continued, while yield gaps have significantly closed for rice and stagnated for wheat. The discrepancy in attainable yield growth between crops reinforces calls for increased agricultural research investment in crops critical to food security^19,30,31^, and suggests region and crop combinations where investment should be targeted. The method introduced here for identifying and analysing yield gaps should be viewed as a complement to computational approaches, particularly those that upscale local and regional results from process-based models^43,44^. The temporal nature of the yield gap analysis presented here provides more insights than can be obtained from a snapshot, and is critical to the definition of the typology categories ‘ceiling pressure’, ‘steady growth’ and ‘stalled floor’, which can help to determine types of intervention and where to target efforts to increase production. Continued growth of the attainable yields identified here will require ongoing investment in agricultural technologies. Assuring this ‘room to grow’ for future crop yields is critical to fulfilling the promise of the green revolution and providing food security for future generations.

## Methods

### Crop data

Tables of annual yield and harvested area data for barley, cassava, maize, oil palm, rapeseed, rice, sorghum, soybean, sugar cane and wheat were compiled following the methods of Ray et al.^56^. In general terms, the data compilation method relies on obtaining and reconciling data for crop yield and area from a variety of public sources. Data are reconciled across administrative units (that is, if the sum of reported state-level production exceeds reported national-level production, the state-level data are reduced by a factor to assure agreement at the coarsest levels). There is a gap-filling procedure that is required when there is a gap in a data series at a subnational level. In this case, the missing data are filled with the last 5 year average data available, so that it scales with the data at the higher administrative level while retaining the subnational patterns for a crop. Full details are available in Ray et al.^56^.

We note that yield data are reported in terms of tonnes per harvested hectare; the same parcel of land can have multiple harvests in a year. A data quality check for each crop–year combination rejected points with harvested area greater than 300% (that is, the multicropping index for the entire region exceeds 3) or yield values greater than 2 s.d. above the area-weighted 97.5th percentile yield value for each crop–year.

Data were developed using the detailed process described in section 1.2 of Supplementary Text 1 in Ray et al.^56^, which in turn is based on the approach of Monfreda et al.^62^. All data are from public sources and can be replicated by a reader using the methods and sources provided. If those sources are no longer available, the second author will provide that data. The maize, rice, wheat and soybean yield data are available in Supplementary Table 8 in Ray et al.^56^. Requests for the actual gridded maps can be sent to the first author of Ray et al.^56^.

### Biophysical data

#### Climate data

We reprocessed global datasets of monthly average temperature and monthly precipitation at 5 arcmin resolution from the WorldClim V2.1 (ref. ^63^) to calculate grids of growing degree days (GDD) with base temperature (*T*_base_) = 0 °C, mean annual precipitation (MAP), precipitation concentration index^64^ (PCI) and a binary vernalization factor (VF) that takes on the value 1 if the coldest monthly winter temperature is less than or equal to 8 °C and zero otherwise. These resulting grids (all at 5 arcmin resolution) were incorporated into data tables as described below.

#### Soils data

We obtained 30 arcsec grids of available water capacity, pH (PH) and soil organic carbon at various soil depths from the SoilGrids1km project^65,66^ (downloaded from www.isric.org on 27 June 2017). After individual depth layers were aggregated to 5 arcmin resolution, soil properties in the top 30 cm were obtained using a trapezoidal integration following Hengl et al.^65^. We included topographical data by downloading 5 arcmin grids with percentage of 100 m × 100 m subpixels with average slopes in the intervals below 10°, between 10° and 30°, and above 30° from Harmonized World Soil Database v 1.2 (ref. ^67^).

These resulting grids (all at 5 arcmin resolution) were incorporated into data tables as described below.

#### Irrigated area

To determine a time series of irrigated area, we scaled the fraction of irrigated area as the maximum proportion of the crop growing area irrigated in each grid cell (IRR) from Mueller et al.^6^ (which was based on MIRCA2000 (ref. ^68^)) using ratios of area equipped for irrigation from Siebert et al.^69^. We use linear extrapolation of the area equipped for irrigation to extrapolate beyond 2005, constraining the result such that IRR is between 0 and 1 (inclusive). These resulting crop-specific grids (all at 5 arcmin resolution) were incorporated into data tables as described below.

#### Data tables

For each combination of administrative unit, crop and year with both yield and area data, we compiled data tables from the gridded yield and area data. The yield and area data for the tables were calculated with an area-weighted average (over the crop-specific harvested area). Similarly, a value for each biophysical parameter in Table 5 was calculated via an area-weighted average over the crop-specific harvested area. All datasets are available at 5 arcmin resolution with the exception of the CRU data that were downscaled from 10 arcmin to 5 arcmin.

**Table 5:** Biophysical variables used in the construction of the yield attainment model

Variable	Definition	Source
GDD	Growing degree days, *T*_base_ = 0°C for all crops	WorldClim V2.1 (ref. ^63^)
MAP	Mean annual precipitation	WorldClim V2.1 (ref. ^63^)
PCI	Precipitation concentration index	WorldClim V2.1 (ref. ^63^), Oliver^64^
IRR	Fraction of area equipped for irrigation	Portmann et al.^68^, Siebert et al.^69^
AWC	Available water capacity in the upper 30 cm of soil	ISRIC^65^
SOC	Soil organic carbon in the upper 30 cm of soil	ISRIC^65^
PH	The pH of the upper 30 cm of soil	ISRIC^65^
SLOPE30	Proportion of area with slope >30°	Harmonized World Soils Database^67^
VF	Vernalization factor, binary used for wheat only; 1 if −8 ≤ *T*_avg_ ≤ 5 °C, where *T*_avg_ is the average temperature of the coldest month	WorldClim V2.1 (ref. ^63^)
*t*	Year	

### Dataset selection

We carried out substantial portions of the analysis with three different datasets: a climatology from WorldClim V2.1 (Fick et al.^63^), a climatology from CRU V4.05 (Harris et al.^70^) and annual climate from CRU V4.05 (Harris et al.^70^). We selected the WorldClim climatology because it led to the lowest temporal cross-validation error, suggesting it is most appropriate for a study focused on the interpretation of time trends. We compared calculations for yield gap trends based on the use of a climatology to annual data to confirm that the conclusions presented in the paper are independent of this choice (Supplementary Table 9).

### Model construction

Quantile regression models were built to predict the 95th quantiles of yield (*Y)* as a linear function of several biophysical input variables at each year. A multistep process was used to assure that model selection artefacts did not introduce spurious time trends. In a first step, we adopted a stepwise approach for selecting a parsimonious model for each crop species. Our starting point was a model including the biophysical input variables shown in Table 5 with terms selected on a physical basis (equation (3) or (4)). For each crop, the parameters of this model were estimated at the 95th quantile from the 5 year time series using the ‘quantreg’ package (version 5.33) in R (version 3.4.0) implementing the method described by Koenker^71^. We carried out this procedure with a non-overlapping windowed dataset smoothed to eight half-decadal intervals via an average over ±2 years. As a step for removing terms, non-significant terms (whose 95% confidence intervals overlap with zero) were sequentially removed (starting with the term whose confidence interval most centrally overlapped 0) until there were no non-significant terms left. The irrigation fraction and irrigation fraction–precipitation cross term were kept in the model regardless of confidence intervals. Then, we further simplified the model using an iterative cross-validation procedure: we generated a series of simplified ‘child’ models by removing each term (excepting linear and quadratic time terms, the irrigation term and the precipitation–irrigation cross term). We sequentially removed each layer of the smoothed time series as a testing dataset, fitted the model on the remaining seven layers and predicted the 95th yield quantile for the removed data. The predictions were assessed by calculating a quantile-regression-specific loss function^71^ based on the difference between the regression model prediction and the yield data for the omitted year. We follow Meinshausen and Ridgeway^72^ and Koenker^71^ to compute the loss function, LF_*⍺*,year_, although we modify equation 3 from Meinshausen and Ridgeway^72^ to allow area weighting, make the summation explicit and add a subscript ‘year’ to denote the central year of each smoothed half decade:

1 $${\rm{LF}}_{\tau ,{\rm{year}}}=\left\{\begin{array}{c}\frac{{\sum }_{i}\tau \left|\,{y}_{i}-{q}_{i}\right|{a}_{i}}{{\sum }_{i}{a}_{i}}\qquad\quad{y}_{i} > {q}_{i}\\ \frac{{\sum }_{i}(1-\tau )\left|\,{y}_{i}-{q}_{i}\right|{a}_{i}}{{\sum }_{i}{a}_{i}}\qquad{y}_{i}\le {q}_{i}\end{array}\right.$$

where LF_*⍺*,year_ is the loss function, *τ* is the quantile (here 0.95), *y*_*i*_ is the set of yield values at each location *i* for ‘year’, *q*_*i*_ is the set of model predictions at each location *i* and *⍺*_*i*_ is the harvested area of the *i*th location in ‘year’ (corresponding to the administrative units used to make up the data tables). These loss functions are summed up for each of the eight half-decadal intervals. The ‘child’ model with the lowest sum of loss functions then becomes the parent model, and the process continues until a model with the lowest loss function is selected as the best quantile regression model. We extended one generation past the lowest loss function in each case to assure there was not a local minimum in that generation.

Thus, a consistent model for each crop was built with the smoothed yield datasets using the cross-validation-in-time procedure discussed above. We then removed time terms from that model and determined model coefficients for each year based on the annual data.

We determined model coefficients for each year with a regularized quantile regression whose loss function was modified to assure that the yield surface encompassed the intended proportion of harvested area. In other words, equation (1) places a weak constraint on the total harvested area above and below the regression surface, so we added a term, *λ*, to enforce this. The regularized quantile regression is shown in equation (2).

2 $$\begin{array}{c}{\rm{LF}}_{\tau ,\rm{year}}= \lambda + \left\{\begin{array}{c}\frac{{\sum }_{i}\tau \left|\,{y}_{i}-{q}_{i}\right|{a}_{i}}{{\sum }_{i}{a}_{i}}\qquad\quad{y}_{i}> {q}_{i}\\ \frac{{\sum }_{i}(1-\tau )\left|\,{y}_{i}-{q}_{i}\right|{a}_{i}}{{\sum }_{i}{a}_{i}}\qquad{y}_{i}\le {q}_{\begin{array}{c}i\\ \end{array}}\end{array}\right.\\ \lambda ={\rm{s.d.}}(y){\sum }_{i}{a}_{i}\left|\frac{{\sum }_{j}{a}_{j}}{\sum a}-\tau \right|\end{array}$$

*i* is summed over all values, and *j* is summed over values for which *q* > *y.*

All results presented in the paper are based on averages of the individual-year version of the model. Single-year results are area-weighted averages over a ±2 year window.

Equation (3) represents all crops other than wheat.

3 $$\begin{array}{l}Y \sim \mathrm{GDD}+\mathrm{MAP}+{\mathrm{GDD}}^{2}+{\mathrm{MAP}}^{2}+\mathrm{GDD}\times\mathrm{MAP}\\ \quad+\,\mathrm{PCI}+\mathrm{PCI}\times \mathrm{MAP}+\mathrm{IRR}+\mathrm{IRR}\times \mathrm{MAP}\\ \quad+\,\mathrm{IRR}\times \mathrm{PCI}+\mathrm{AWC}+\mathrm{SOC}+\mathrm{Slope}\,30+\mathrm{PH}\end{array}$$

Equation (4) represents only wheat.

4 $$\begin{array}{l}Y \sim \mathrm{GDD}+\mathrm{MAP}+{\mathrm{GDD}}^{2}+{\mathrm{MAP}}^{2}+\mathrm{GDD}\times \mathrm{MAP}+\mathrm{PCI}+\mathrm{PCI}\\ \quad\,\times \mathrm{MAP}+\mathrm{IRR}+\mathrm{IRR}\times \mathrm{MAP}+\mathrm{IRR}\times \mathrm{PCI}+\mathrm{AWC}\\ \quad\,+\,\mathrm{SOC}+{\mathrm{Slope}}\,30+\mathrm{PH}+\mathrm{VF}+\mathrm{VF}\times \mathrm{GDD}+\mathrm{VF}\times \mathrm{MAP}\end{array}$$

### Selected quantile regression models for 95th percentile yield

Table 6 represents the terms included in each crop-specific selected model.

**Table 6:** Terms included in each selected model

Barley	Y∼1+MAP+GDD^2+PCI+IRR+IRR*MAP+IRR*PCI+SLOPE30
Cassava	Y∼1+GDD+MAP^2+GDD^2+IRR+IRR*MAP
Maize	Y∼1+GDD+MAP+GDD^2+PCI+IRR+IRR*MAP+SOC+SLOPE30+PH
Oil palm	Y∼1+GDD+MAP+GDD*MAP+GDD^2+PCI+IRR+IRR*MAP+SOC
Rapeseed	Y∼1+GDD+MAP+GDD*MAP+IRR+IRR*MAP+IRR*PCI+SLOPE30
Rice	Y∼1+GDD+MAP+GDD*MAP+MAP^2+GDD^2+IRR+IRR*MAP+MAP*PCI+IRR*PCI+SOC+PH
Sorghum	Y∼1+GDD+MAP+IRR+IRR*MAP+MAP*PCI+IRR*PCI+SLOPE30
Soybean	Y∼1+MAP+MAP^2+PCI+IRR+IRR*MAP+IRR*PCI+PH
Sugar cane	Y∼1+MAP+MAP^2+GDD^2+IRR+IRR*MAP+PH
Wheat	Y∼1+GDD+GDD*MAP+MAP^2+IRR+IRR*MAP+MAP*PCI+VF*GDD+VF+SLOPE30+PH

### Model coefficients

Model coefficients are given in the Supplementary Information and Supplementary Table 14. The calculations are carried out using *z*-scores, so the model coefficients are unitless. Supplementary Table 14 contains the normalization factors relating the *z*-scores to the variables in physical units.

### Model outputs

#### Overview

Model predictions and their confidence intervals were derived with a bootstrap method. Using 1,000 random samples (with replacement) of the crop and biophysical data, we generated 1,000 realizations of the coefficients of the quantile regression model for each crop. These 1,000 realizations were used to determine the confidence intervals. We used 95th percentile confidence intervals unless noted otherwise.

#### Calculation of time to yield gap closure

Calculation of time to yield gap closure is based on a linear regression of annual yield gaps over the interval 1998–2012 (to assess data circa 2000–circa 2010.) We perform a linear regression on each of the 1,000 time series of yield gap at each political unit. If the 2.5th percentile and 97.5th percentile linear slopes have the same sign, the time to yield gap closure is considered significant. For each of the 1,000 realizations, the zero-crossing year of the average linear fit is at 1998 −*x*0/*x*1, where *x*0 is the *y*-value at 1998 and *x*1 is the slope. Thus, yield gap closure time *t*_close_ relative to 2010 is calculated as ${t}_{\mathrm{close}}=-\frac{{x}_{0}}{{x}_{1}}-12$ for each realization. If, for a political unit, the time to yield gap closure is considered significant, the median closure time is used for analysis.

The calculation used to construct Table 4 uses the same procedure, but over the time period 1986–2000.

#### Calculation of year-specific data

All data are computed as a ±2 year average around the year presented, with the an area-weighted average carried out over five individual-year calculations of the specific quantity. As an example, to determine the circa 2010 global average yield gap, the attainable yield surfaces are calculated for 2008, 2009, 2010, 2011 and 2012; then, the actual yield surfaces are subtracted from these for each year, and the five resulting maps of yield gap are averaged together with weights according to the reported harvested area in each year.

#### Calculation of stagnation probabilities

To identify yield stagnation, we carried out a piecewise linear regression over 27 years of data, from 1986 to 2012, with a single discontinuity in slope at 2000. For each crop, at each political unit, we consider stagnation to occur in the following case: 95th percentile confidence limits around the 1986–2000 slope are both positive, and lower confidence limits around the 2000–2012 slope are negative. This definition of stagnation follows Grassini^14^. These test years were chosen so that there are 15 years in the time series leading to 2000, allowing us to draw conclusions from trends in yield gap from the 15 year time series from 1998 to 2012.

#### Calculation of impact of yield gap closure on likelihood of stagnation

To assess the impact that yield gap closure trends on the likelihood of stagnation, we assessed linear yield gap closure trends over a 15 year time series from 1986 to 2000 (Supplementary Table 7). We found that linear yield gap closure trends that will be closing within 30 years are associated with a doubling of the probability that the yield series from 2000 to 2012 will then stagnate (using the definition of stagnation in a previous paragraph). To assure that this result is not an artefact of quick rise in yield making a finding of stagnation more likely, we compared stagnation likelihood across political units that were in the third quartile of yield growth rates and had yield gaps that were on a trajectory to close within 30 years, and political units that were in the third quartile of yield growth rates. We repeated this analysis with a 75th percentile confidence interval to test the sensitivity of the result to the number of census units identified as undergoing yield stagnation (Supplementary Table 8).

### Reporting summary

Further information on research design is available in the Nature Portfolio Reporting Summary linked to this article.

### Supplementary information

Supplementary Information Supplementary Figs. 1–6, Tables 1–9 and Methods.

Reporting Summary

Supplementary Tables Supplementary Tables 10–24.

## Data Availability

All weather, soil and irrigation data used in this study are publicly available and sourced in refs. ^63,65–70^ of Methods. Crop yield and area data are derived from publicly available sources (agricultural census and survey reports as identified in ref. ^56^) as described and further referenced in Methods. All data generated in the current study (Figs. 1, 2 and 4) as well as annual potential yield surfaces for ten crops over the period 1973–2012 can be downloaded from 10.5281/zenodo.10234041. All data inputs to the study, as well as all results, are available upon request from the corresponding author. The authors commit to full and timely cooperation with any validation studies.

## Code availability

The code was developed in the Matlab and R programming languages and can be downloaded from 10.5281/zenodo.10234041.
