# Supplementary material for: Global spatially explicit yield gap time trends reveal regions at risk of future crop yield stagnation
Source: Nat Food. 2024 Jan 26;5(2):125–35. doi: 10.1038/s43016-023-00913-8 (PMC10896731; doi:10.1038/s43016-023-00913-8)
Supplement: Supplementary file 1 — Supplementary Figs. 1–6, Tables 1–9 and Methods. [file 43016_2023_913_MOESM1_ESM.pdf]

nature food

Article

https://doi.org/10.1038/s43016-023-00913-8

Global spatially explicit yield gap time trends reveal regions at risk of future crop yield stagnation

In the format provided by the authors and unedited

Supplementary Information

Global spatially explicit yield gap time trends reveal regions at risk of future crop yield stagnation

J. S. Gerber et al

Nature Food

Table of Contents

FIGURE S1 GROWTH IN ATTAINABLE YIELD (MAPS) 2

FIGURE S2 MAPS OF LINEAR GROWTH IN YIELD GAP CIRCA 1975-2010 3

FIGURE S3 TIME TO CLOSURE OF YIELD GAPS (FIGURE 2 OF MAIN TEXT) 4

FIGURE S4 YIELD GAP TYPOLOGY MAPS (FIGURE 4 OF MAIN TEXT) 5

FIGURE S5 COUNTRY-SPECIFIC TIMESERIES OF ACTUAL AND ATTAINABLE YIELD 6

FIGURE S6 COMPARISON TO RESULTS GLOBAL AGRO ECOLOGICAL ZONES (GAEZ) 11

TABLE S1 PERCENT OF HARVESTED AREA WITH GROWTH IN ATTAINABLE YIELD. 12

TABLE S2 AVERAGE ANNUAL PERCENT GROWTH IN ATTAINABLE YIELD. 12

TABLE S3 FIXED-AREA COUNTERFACTUAL TABLES 13

TABLE S4A AREA WITH SIGNIFICANT YIELD GAP INCREASE 14

TABLE S4B AREA WITH SIGNIFICANT YIELD GAP DECREASE 15

TABLE S5 LIKELIHOOD OF STAGNATION AFTER OBSERVED YIELD GAP CLOSURE 15

DEFINITION OF YIELD GAP CLOSURE TYPOLOGIES 16

CEILING PRESSURE CALCULATIONS 17

FIGURE S4 – CALCULATION OF TIME TO YIELD GAP CLOSURE 18

YIELD STAGNATION ASSESSMENT 18

DEFINITION OF REGIONS 22

DATASET SELECTION 22

REFERENCES 25

Figure S1 Growth in attainable yield (maps)

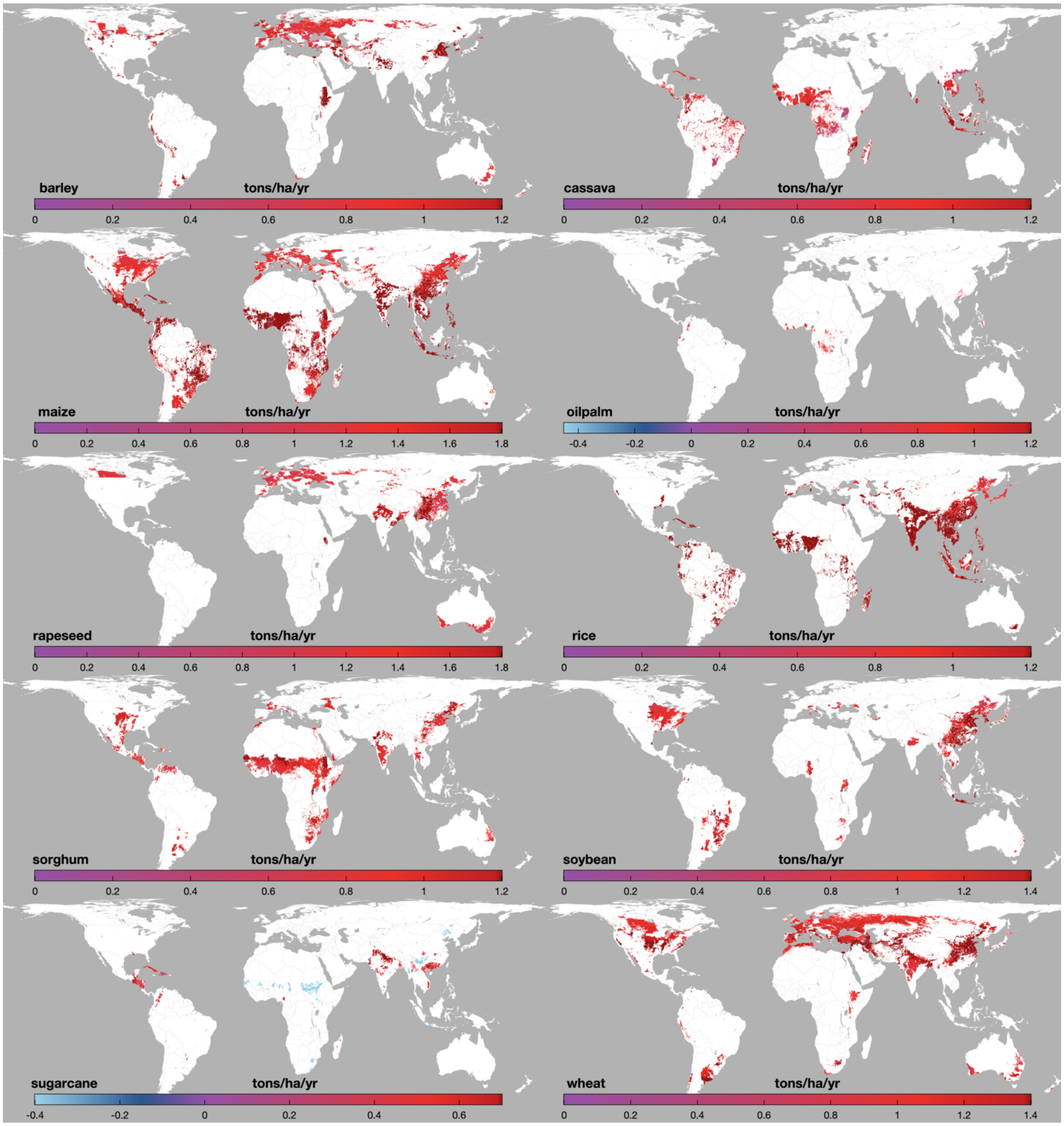

Fig S1. Percent growth in yield-ceiling calculated within each political unit relative to year 2000. In each political unit, linear growth is calculated for multiple realizations from 1975 to 2010. Average linear growth rate is plotted here as annual percent change relative to 2000.

Figure S2 Maps of linear growth in yield gap circa 1975-2010

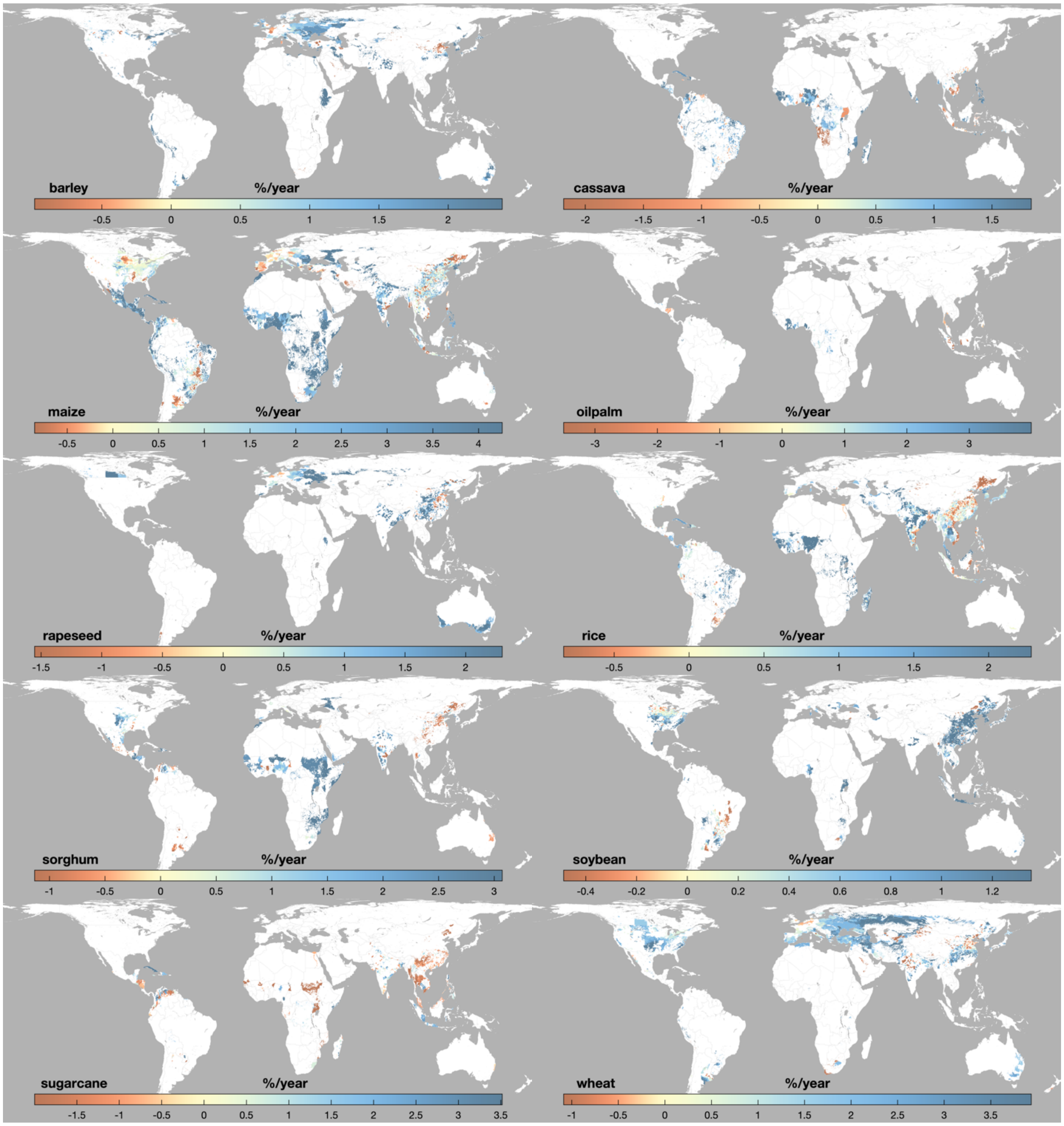

Fig S2 Linear yield gap trend from circa 1975 to circa 2010 expressed as annual percentage change relative to circa year 2000 yield

Figure S3 Time to closure of yield gaps (Figure 2 of main text)

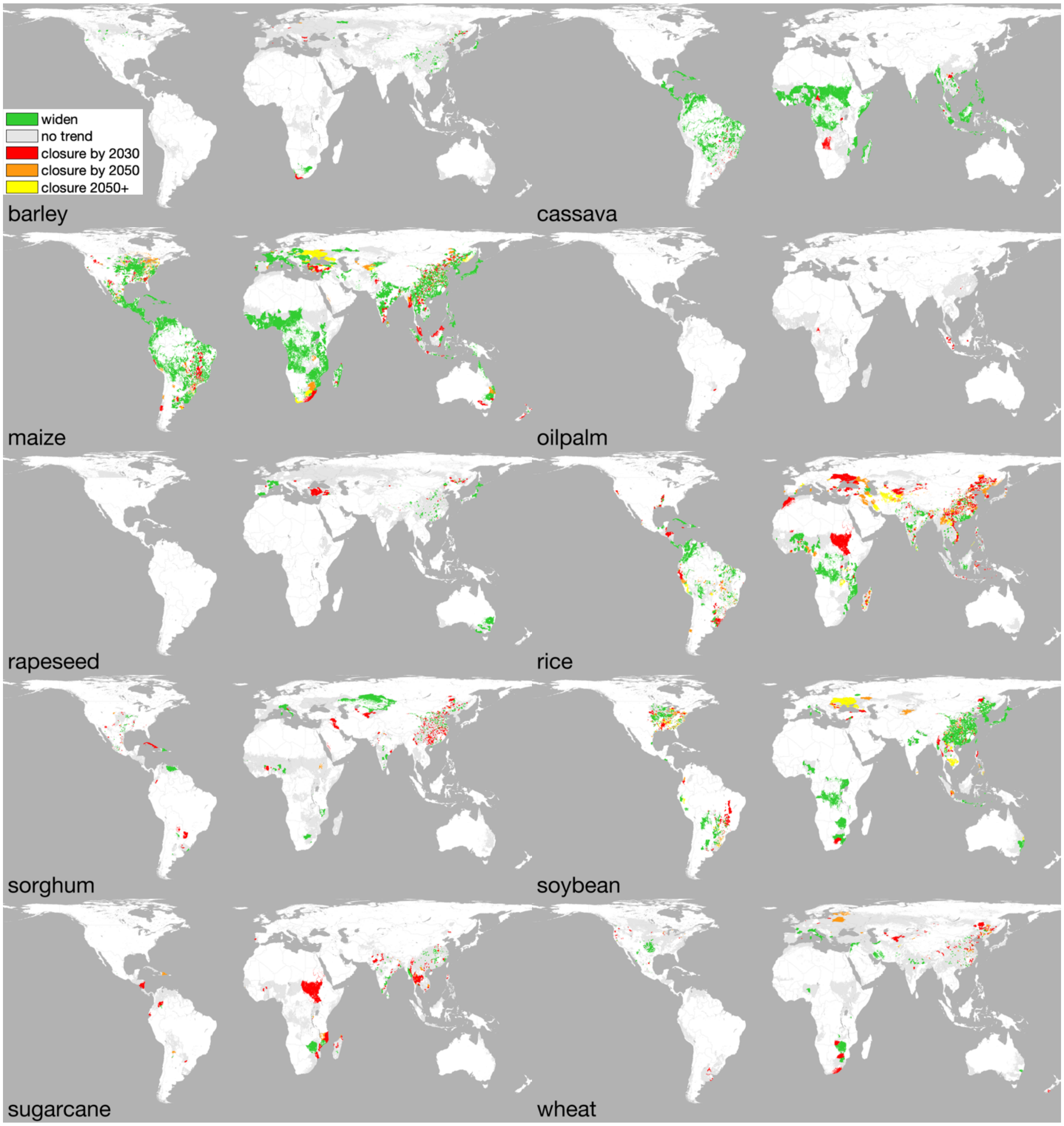

Figure S3 Time to closure of yield gaps (i.e., zero yield gap) based on linear extrapolation of trends from circa 2000 to circa 2010 for maize, rice, soybean and wheat. Yield gap closure time is defined as the crossing point of linear trend of attainable yield and linear trend of actual yield relative to year 2010. Light gray color "no trend" indicates that no yield gap closure occurs within 95th percentile confidence intervals. Green color ("widen") indicates that yield ceiling and linear trend are significantly diverging (i.e. crossing point is prior to the year 2010)

Figure S4 Yield Gap Typology maps (Figure 4 of main text)

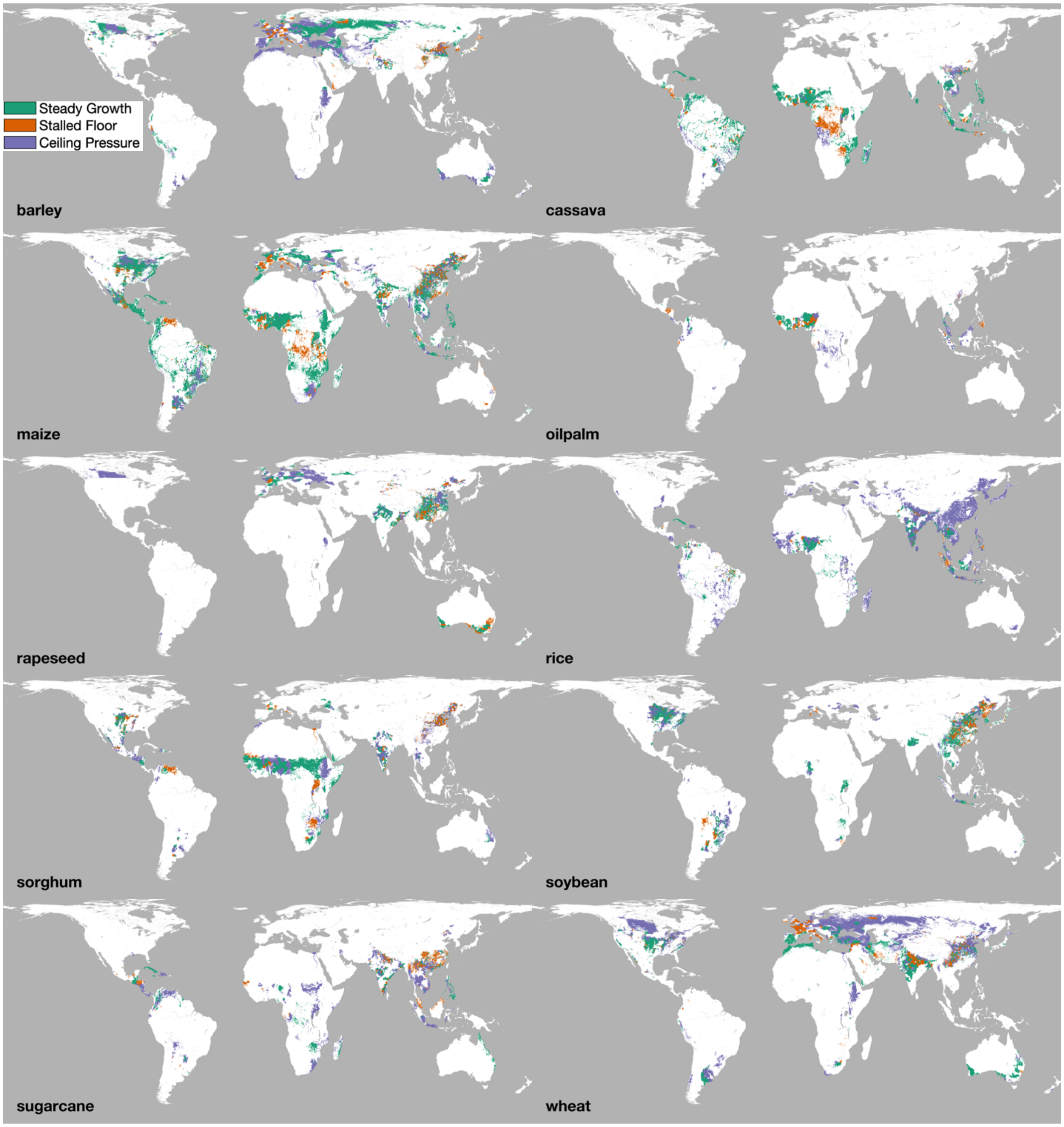

Figure S4 Typologies of yield gap change relative to interval from circa 2000 to circa 2010. Legend uses a schematic representation of the three typologies ("Steady Growth","Stalled Floor","Ceiling Pressure").

Figure S5 Country-specific timeseries of actual and attainable yield

Figs S5a Steady Growth Examples

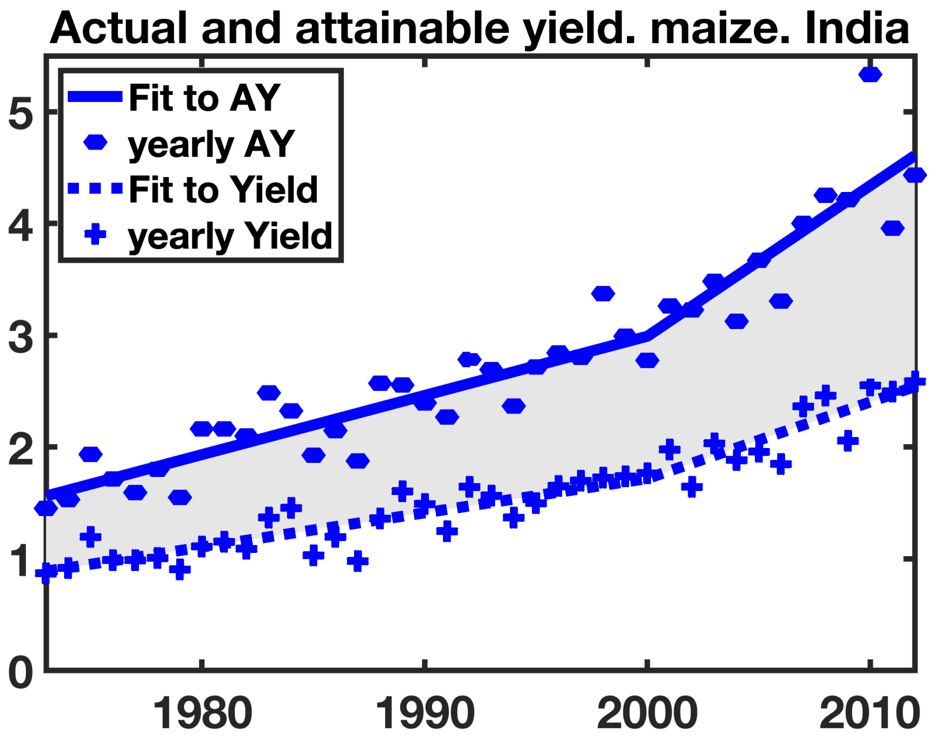

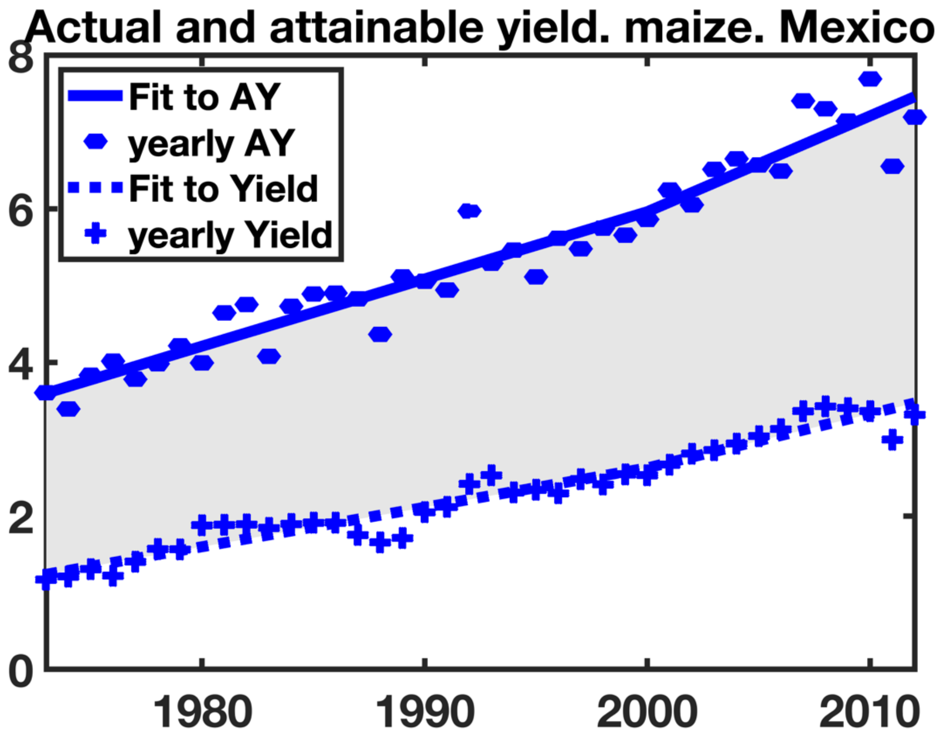

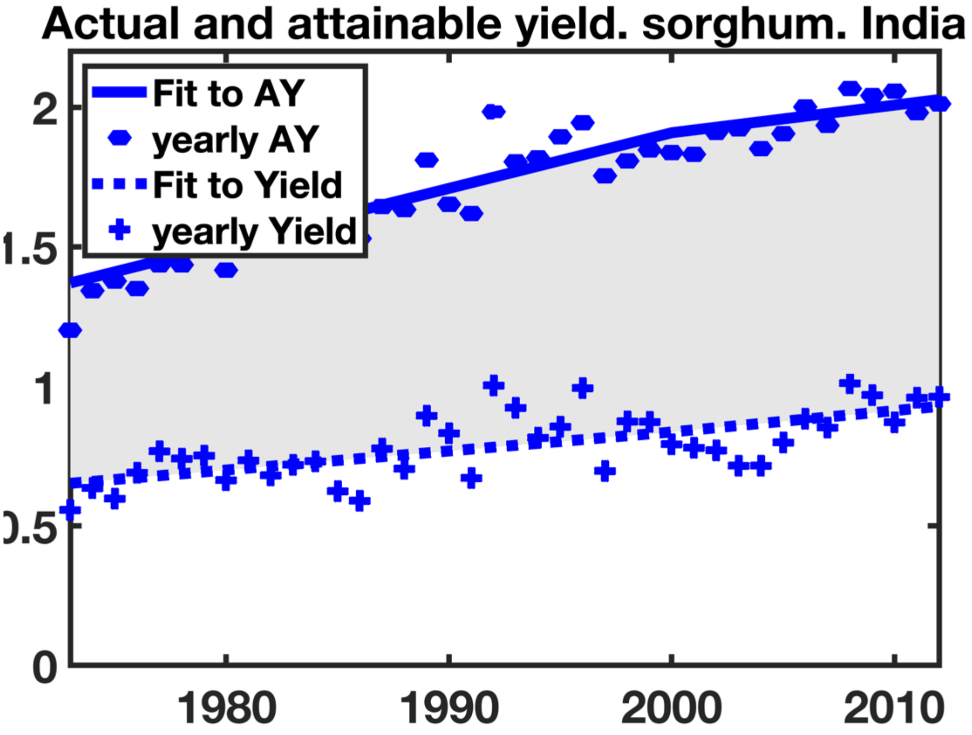

Figs S5b Stalled Floor examples

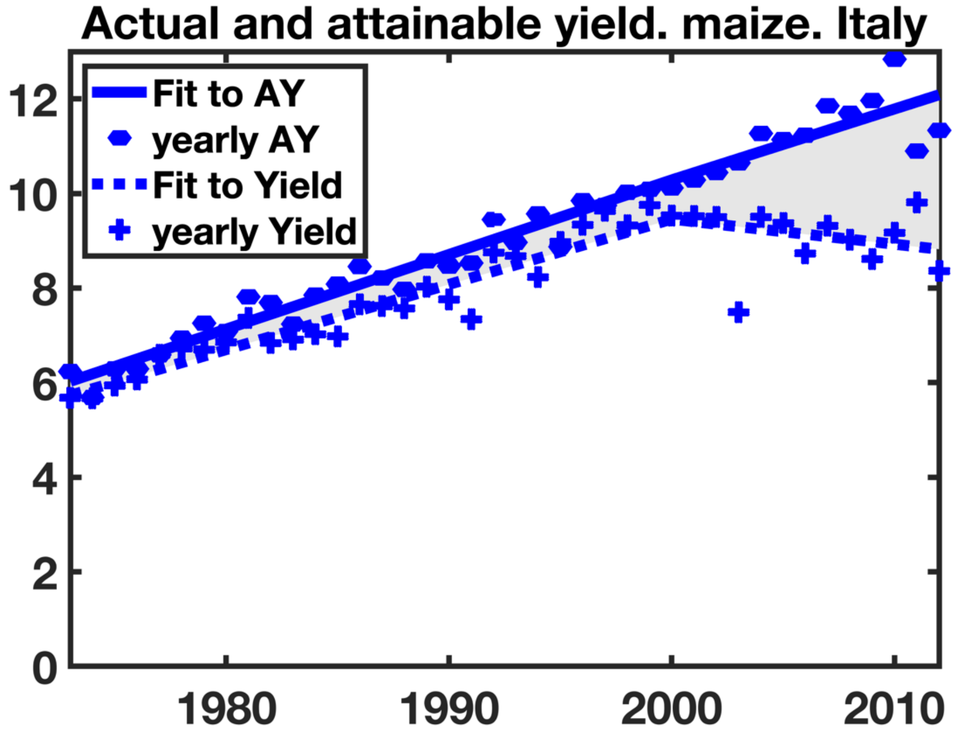

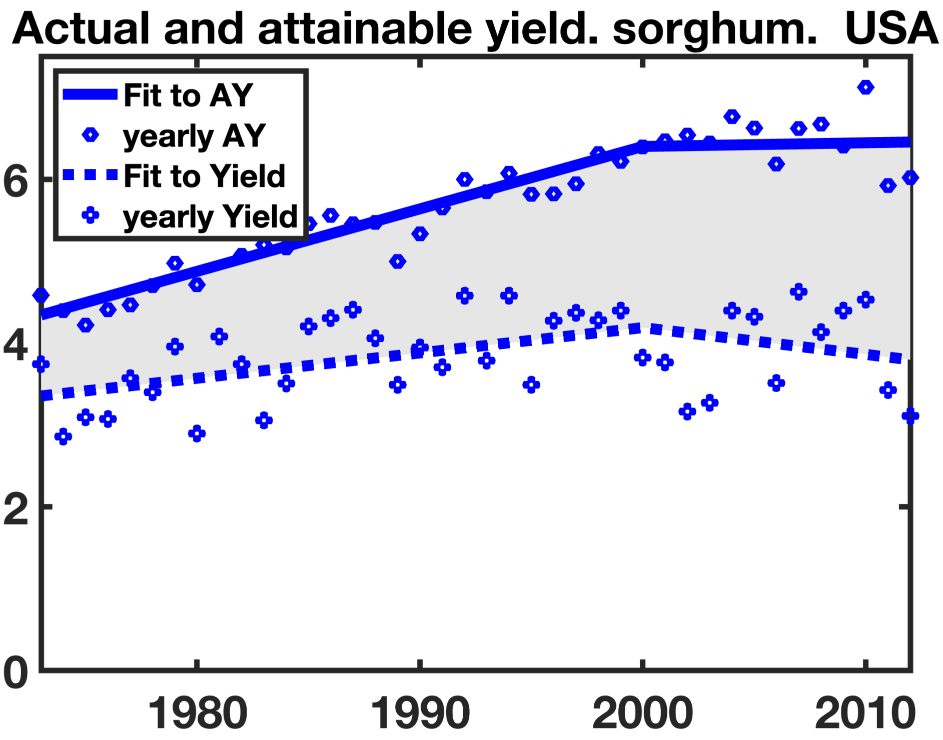

Figs S5c Ceiling Pressure examples

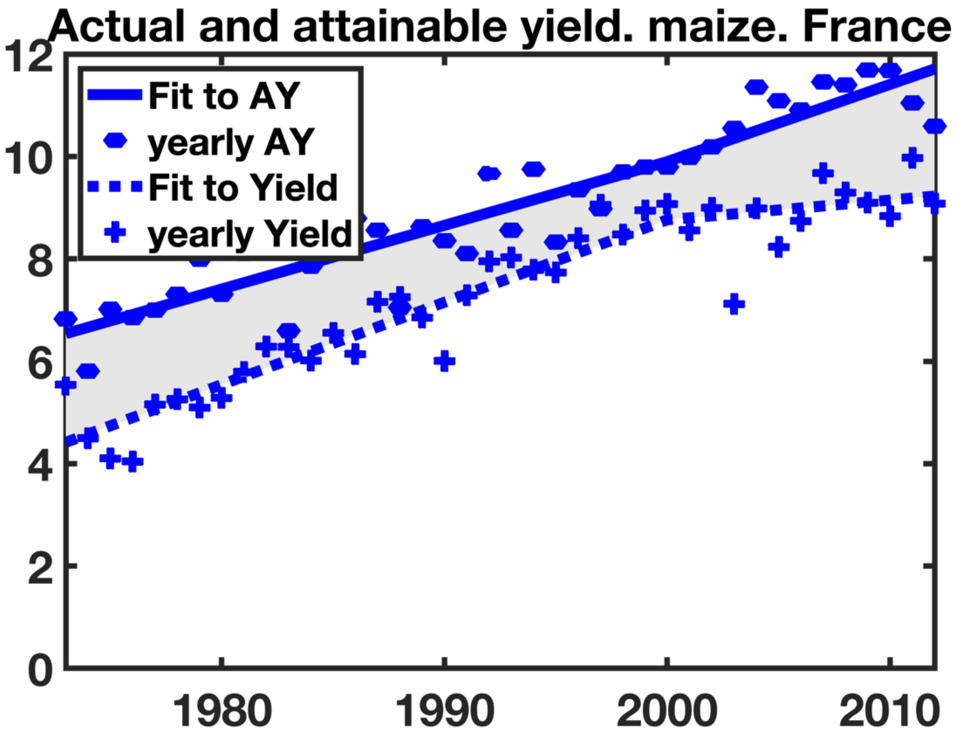
Actual and attainable yield. maize. France
Fit to AY
yearly AY
Fit to Yield
yearly Yield
0
2
4
6
8
10
12
1980
1990
2000
2010

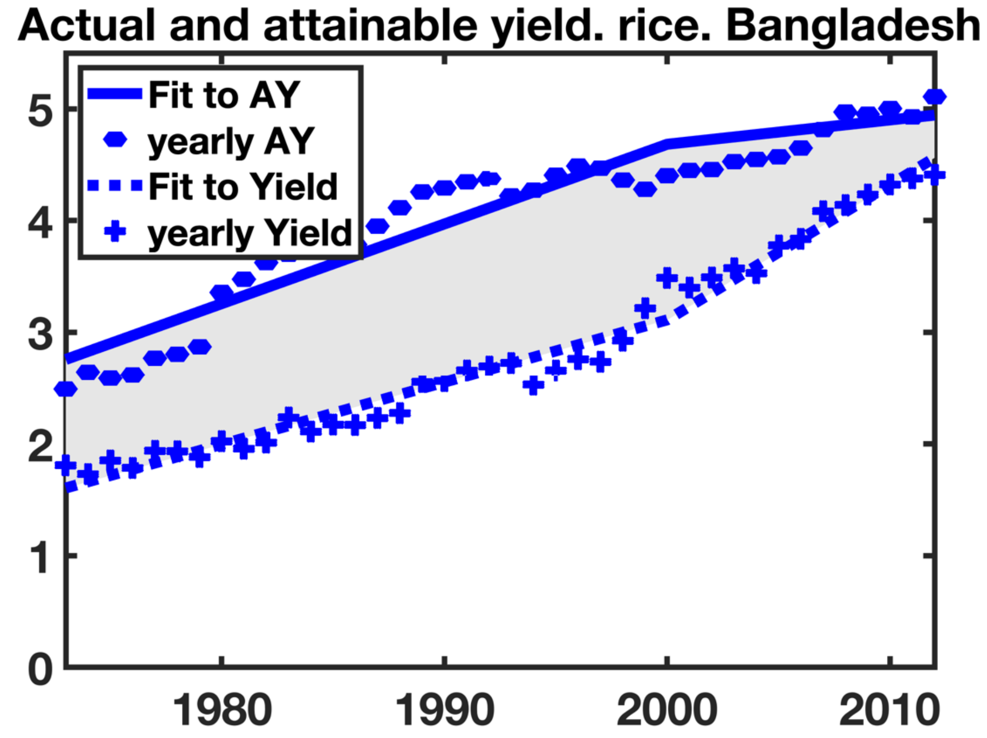
Actual and attainable yield. rice. Bangladesh
Fit to AY
yearly AY
Fit to Yield
yearly Yield
0
1
2
3
4
5
1980
1990
2000
2010

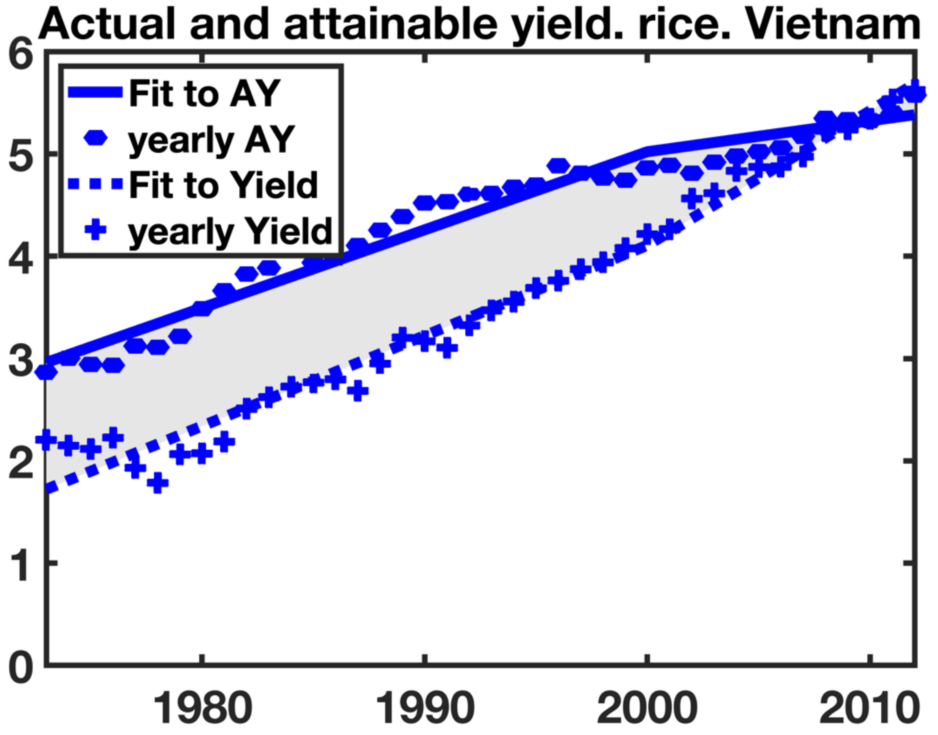

Actual and attainable yield. rice. Vietnam
Fit to AY
yearly AY
Fit to Yield
yearly Yield
0
1
2
3
4
5
6
1980
1990
2000
2010

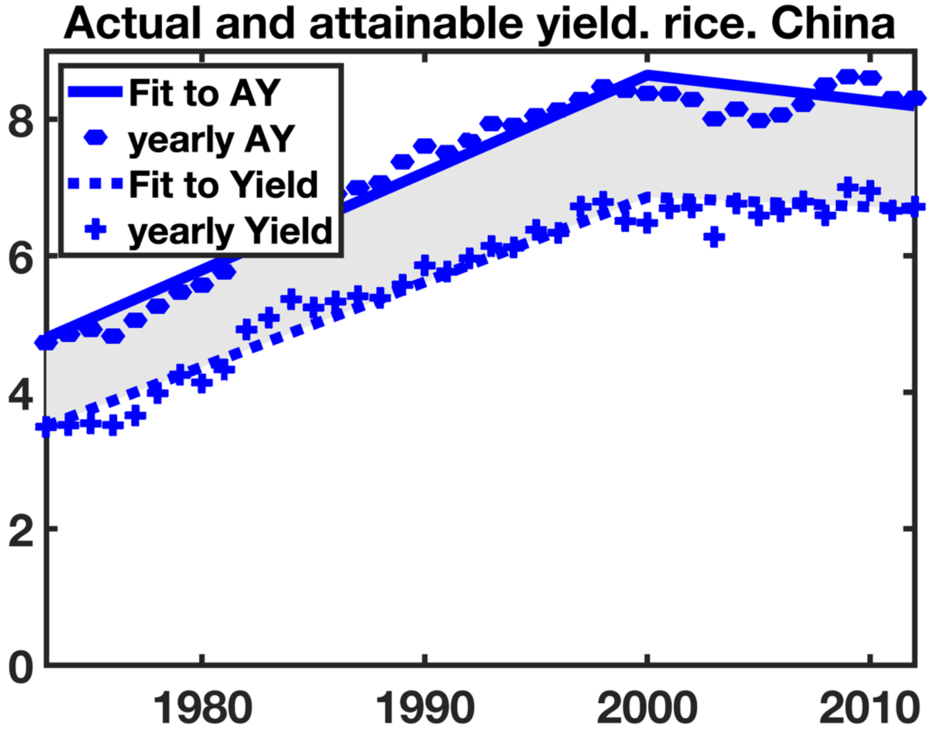

Actual and attainable yield. rice. China
Fit to AY
yearly AY
Fit to Yield
yearly Yield
0
2
4
6
8
1980
1990
2000
2010

Figure S6 Comparison to results Global Agro Ecological Zones (GAEZ)

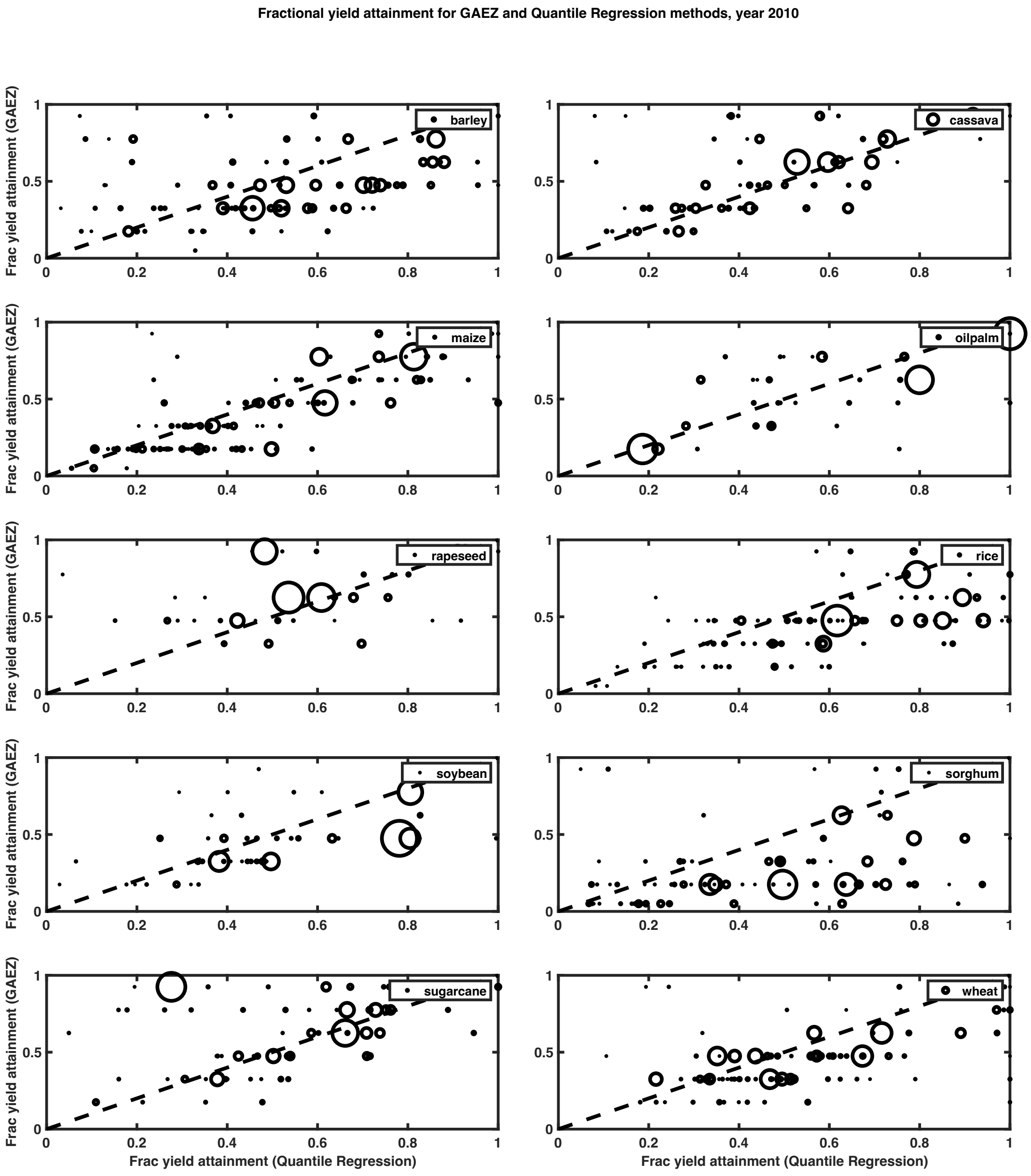

Figure S6 Fractional yield attainment as calculated with the quantile regression method and reported by GAEZ (Fischer, G. 2000) *Each data point represents a country, area of plot markers is proportional to harvested area of that country. GAEZ fractional yield attainment is plotted as the central value of the reported categories.*

Table S1 Percent of harvested area with growth in attainable yield. *Areas for which attainable yields increase over the indicated period with 95% confidence intervals. Area calculation is based on nominal starting year of interval except for 1975-2010 interval which is based on year 2000 area.*

	Entire period	Decades			
	**1975-2010**	**1975-1985**	**1980-1990**	**1990-2000**	**2000-2010**
**barley**	69%	43%	37%	0%	1%
**cassava**	85%	0%	0%	0%	75%
**maize**	100%	88%	85%	98%	97%
**oil palm**	7%	0%	0%	0%	1%
**rapeseed**	99%	86%	68%	0%	1%
**rice**	100%	94%	99%	48%	65%
**sorghum**	94%	53%	10%	10%	0%
**soybean**	100%	89%	91%	85%	86%
**sugarcane**	56%	6%	0%	4%	0%
**wheat**	98%	78%	67%	17%	4%

Table S2 Average annual percent growth in attainable yield. *Reported growth is per-year linear growth relative to nominal starting year of interval (except for 1975-2010 interval which is calculated relative to year 2000 yield gap to facilitate comparison with 2000-2010 interval.). Square brackets [ ] indicate not significant (95% confidence intervals encompass zero).*

	Entire period	Decades			
	**1975-2010**	**1975-1985**	**1980-1990**	**1990-2000**	**2000-2010**
**barley**	0.8%	1.3%	[1.1%]	[0.5%]	[0.7%]
**cassava**	0.8%	[0.1%]	[0.0%]	[0.6%]	3.4%
**maize**	1.4%	1.4%	0.9%	1.9%	1.5%
**oil palm**	1.3%	[1.6%]	[1.4%]	[1.2%]	[0.3%]
**rapeseed**	1.6%	2.2%	1.8%	1.2%	2.1%
**rice**	1.2%	1.8%	1.8%	0.6%	0.7%
**sorghum**	[0.1%]	[0.5%]	[-0.5%]	[0.5%]	0.3%
**soybean**	1.1%	1.1%	1.0%	[1.3%]	1.0%
**sugarcane**	0.4%	[0.6%]	[0.2%]	[0.7%]	[-0.2%]
**wheat**	1.3%	1.8%	[1.7%]	[0.8%]	0.7%

Table S3 Fixed-area counterfactual tables

Table** S3a* ***Growth in attainable yields with fixed area counterfactual. *Area with significant growth in attainable yields calculated as area-weighted percentage of circa 2000 political units for which attainable yields increase over the indicated time period with 95% probability. Average annual percent growth calculations are relative to circa year 2000 attainable yields. Square brackets [ ] indicate not significant (95% confidence intervals include zero).*

	Average annual percent growth in attainable yield (area-weighted)		Average annual percent growth in attainable yield (area-fixed counterfactual)	
	**1975-2010**	**2000-2010**	**1975-2010**	**2000-2010**
**barley**	0.8%	[0.7%]	0.9%	[0.6%]
**cassava**	0.8%	3.4%	0.8%	3.4%
**maize**	1.4%	1.5%	1.5%	1.7%
**oil palm**	1.3%	[0.3%]	[0.3%]	[-0.2%]
**rapeseed**	1.6%	2.1%	1.3%	1.5%
**rice**	1.2%	0.7%	1.3%	0.8%
**sorghum**	[0.1%]	0.3%	1.0%	[0.8%]
**soybean**	1.1%	1.0%	1.1%	1.1%
**sugarcane**	0.4%	[-0.2%]	0.3%	[-0.2%]
**wheat**	1.3%	[0.7%]	1.3%	[0.7%]

Table** S3b* ***Tends in yield gaps with fixed area counterfactual. *Average annual percent change in globally averaged yield gaps. Reported change is per-year linear change relative to year 2000 yield. Square brackets [ ] indicate not significant (95% confidence intervals encompass zero). Means are calculated after rejecting outliers (some realizations have yield gap ~ 0, leading to infinite relative growth rates). Outliers are defined as points outside of the interval mean+4*std.*

	Average annual trend (percent) in yield gap (area-weighted)		Average annual trend (percent) in yield gap (area-fixed counterfactual)	
	**1975-2010**	**2000-2010**	**1975-2010**	**2000-2010**
**barley**	0.9%	[0.3%]	0.9%	[0.1%]
**cassava**	[0.6%]	5.6%	[0.6%]	4.9%
**maize**	1.2%	1.6%	1.3%	1.8%
**oil palm**	[-0.4%]	[-2.6%]	[-0.4%]	-1.6%
**rapeseed**	1.5%	1.5%	1.0%	[0.7%]
**rice**	0.8%	[-0.4%]	0.8%	[-0.3%]
**sorghum**	[0.4%]	[0.8%]	1.2%	[0.8%]
**soybean**	0.8%	1.4%	1.0%	1.4%
**sugarcane**	[0.1%]	[-1.3%]	[0.1%]	[-0.9%]
**wheat**	1.2%	[0.1%]	1.3%	[0.1%]

Table S3c Changes in yield ceiling and yield gap compared to fixed-area counterfactual

	Percent change in yield ceiling compared to fixed-area counterfactual, 1975-2010	Percent change in yield gap compared to fixed-area counterfactual, 1975-2010
**barley**	-6.60%	-4.50%
**cassava**	-0.50%	-7.30%
**maize**	-3.10%	-3.20%
**oil palm**	32.50%	-47.30%
**rapeseed**	12.10%	15.00%
**rice**	-4.40%	-0.40%
**sorghum**	-47.50%	-22.10%
**soybean**	-5.20%	-20.10%
**sugarcane**	2.70%	-3.50%
**wheat**	-2.20%	-10.20%

Table S4a Area with significant yield gap increase. *Area calculation based on starting year of interval except for 1975-2010 interval which is based on year 2000 area.*

	Entire period	Decades			
	**1975-2010**	**1975-1985**	**1980-1990**	**1990-2000**	**2000-2010**
**barley**	36%	21%	6%	4%	1%
**cassava**	48%	0%	3%	16%	46%
**maize**	71%	41%	45%	58%	55%
**oil palm**	10%	0%	0%	1%	0%
**rapeseed**	52%	29%	29%	5%	5%
**rice**	56%	58%	63%	21%	15%
**sorghum**	62%	28%	16%	11%	5%
**soybean**	61%	44%	48%	42%	37%
**sugarcane**	28%	6%	4%	17%	4%
**wheat**	62%	36%	31%	8%	5%

Table S4b Area with significant yield gap decrease. *Area calculation based on starting year of interval except for 1975-2010 interval which is based on year 2000 area.*

	Entire period	Decades			
	**1975-2010**	**1975-1985**	**1980-1990**	**1990-2000**	**2000-2010**
**barley**	4%	1%	1%	1%	1%
**cassava**	13%	15%	15%	10%	3%
**maize**	16%	14%	14%	15%	14%
**oil palm**	7%	0%	0%	0%	4%
**rapeseed**	5%	3%	3%	5%	1%
**rice**	24%	9%	8%	25%	24%
**sorghum**	7%	8%	7%	3%	2%
**soybean**	15%	10%	10%	21%	17%
**sugarcane**	22%	3%	4%	6%	13%
**wheat**	8%	1%	2%	1%	4%

Table S5 Likelihood of stagnation after observed yield gap closure

Table S5 *Likelihood of stagnation after year 2000 based on prior 15 years of yield gap trend in conjunction with other conditions Here, stagnation occurs if a bi-linear fit to yield has a slope greater than 0 before 2000 (lower 95th percentile confidence interval > 0) and less than 0 after 2000 (lower 95th percentile confidence interval < 0). Details in methods section.*

	**All census units**	**Census units with yield gaps trending to close within 20 years**	**Census units with slope of actual yield in top quartile**	**Census units with slope of actual yield in top quartile AND yield gaps trending to close within 20 years**
	Percentage of census units where bi-linear fit of yield timeseries shows stagnation	*Percentage of census units with stagnation AND where linear fit to yield gaps over interval 1986-2000 has significantly negative slope and median yield gap closure time of less than 20 years*	*Percentage of census units in the top quartile of values of slope of actual yield (linear fit) with stagnation*	*Percentage of census units in the top quartile of slope values AND median yield gap closure less than 20 yrs with stagnation*
**barley**	34.6%	68.0%	59.5%	68.3%
**cassava**	14.2%	42.4%	24.0%	40.0%
**maize**	17.0%	42.5%	19.7%	46.6%
**oil palm**	63.8%	NA	72.6%	NA
**rapeseed**	16.0%	45.9%	26.8%	47.5%
**rice**	25.5%	54.9%	41.1%	61.3%

**sorghum**	29.8%	86.6%	57.2%	92.6%
**soybean**	15.6%	58.5%	26.3%	61.8%
**sugarcane**	32.3%	57.6%	52.0%	60.2%
**wheat**	19.1%	56.0%	32.9%	58.7%

Definition of yield gap closure typologies

Yield gap closure typologies are described in the table below. Confidence intervals on slopes of bilinear fit to yield are the 95th percentile confidence intervals calculated by the regress function in Matlab. Confidence intervals on slopes of bilinear fit to attainable yield are determined by first creating an ensemble of ordinary least squares fits of a bilinear function to each of 1000 realizations of attainable yield, then calculating values of the 0.025th and 0.975th quantiles of ensembles of slopes.

Table S6 – Typologies of yield gap closure (Figure 4 in manuscript) with exact definition of typologies.

<table>
<tr><th>Name</th><th>Illustration</th><th>Archetypical interpretation</th><th>Category definition</th></tr>
<tr><td>Steady growth
Yield gap increasing, Ceiling increasing Floor increasing.</td><td>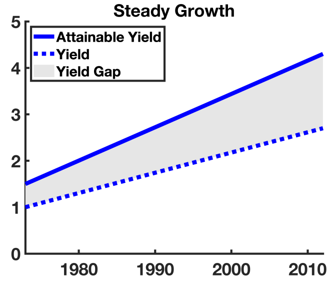
</td><td>Benefits of agronomic research: Yield gap is growing because attainable yield (“ceiling”) and actual yield (“floor”) benefit from agronomic investment in new technologies and increased uptake of management practices.</td><td>Slope of post 2000 yield gap is positive AND slope of bi-linear fit to attainable yield does not undergo a significant change before and after 2000 AND slope of bi-linear fit to actual yield does not undergo a significant change before and after 2000</td></tr>
<tr><td>Stalled floor
Yield gap increasing, Ceiling increasing Floor shows significant slowdown</td><td>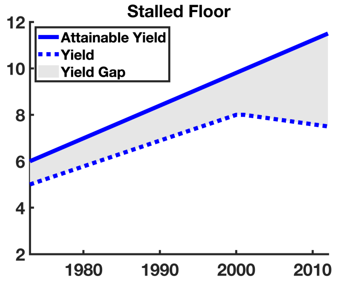
</td><td>Lack of uptake of yield-maximizing practices. Yield gap is increasing because “best in class” management practices for maximizing yield are not widely adopted for a number of reasons including economic barriers, selection of lower-yielding higher-quality cultivars, or adoption of environmental policies.</td><td>Slope of post 2000 yield gap is positive AND slope of bi-linear fit to actual yield does undergo a significant change before and after 2000</td></tr>
</table>

**Ceiling Pressure** Yield gap decreases and/or Ceiling growth slowdown	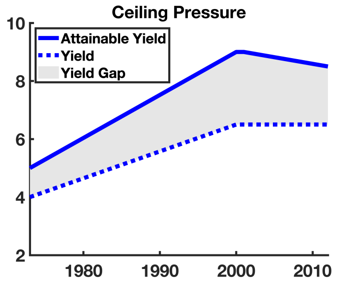	**Need for agronomic research:** Small yield gaps indicate a need for breeding and "new agronomy" to improve yield ceilings.[24]	**Slope of post 2000 yield gap is negative**<br>**OR slope of bi-linear fit to attainable yield does undergo a significant decrease before and after 2000**

Ceiling Pressure Calculations

To assess the relationship between trends in yield gap closure and future yield stagnation we first assessed yield gap trends from 1986 to 2000, and separately calculated yield stagnation subsequent to year 2000 based on the entire time-series from 1973-2012. The reason for these differing time periods is that we wish to assess the utility of a 15 year time-series of Yield Gap for predicting future yield stagnation.

Yield gap trends and closure: We calculated yield gap closure from 1986 to 2000 for each census unit for each crop as follows: we calculated the slope of the linear best fit of actual yield data, and a linear best fit for each N=1000 realizations of attainable yield (AY). This is shown for a particular case in Figure S1 (where only 20 AY fits are shown.) For each of the AY linear fits, a yield gap closure time is the time at which the AY linear fit intersects the linear fit of actual yield. If the 2.5th and 97.5th percentile values of the resulting distribution of yield gap closure times are greater than the year 2000, the yield gap closure is considered significant, and the median yield gap closure time is used for comparison.

Figure S4 – Calculation of time to yield gap closure

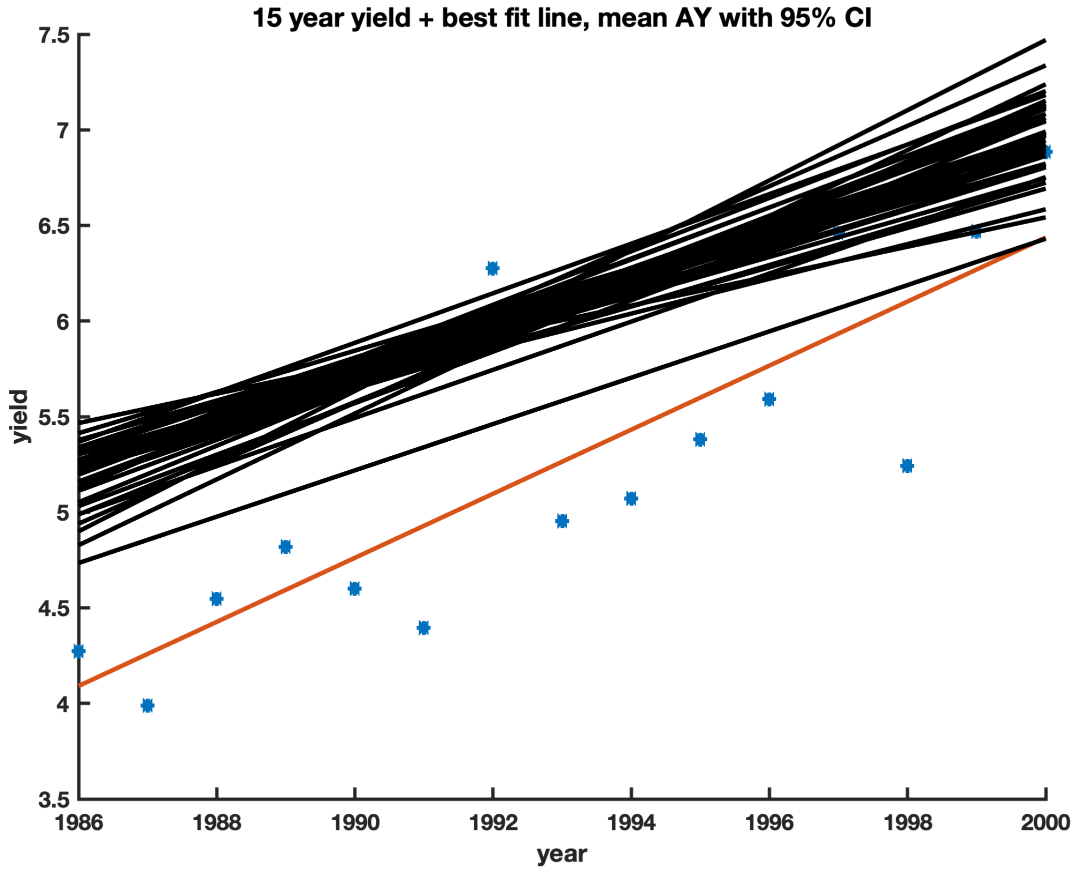

*Figure S4 Example of construction of curves used to calculate yield gap closure time. Blue * represent actual yield data, red solid line is linear least squares best fit, 20 linear fits to attainable yield realizations is also shown. Intersections (not shown) between line representing linear fit to actual yield and lines representing linear fit to attainable yield are 'yield gap closure' X-axis points of these intersections are yield gap closure dates, difference from year 2000 is yield gap closure time.*

Yield stagnation assessment

To assess yield stagnation, we performed a bi-linear regression over the entire timeseries with a discontinuity in slope at year 2000 for each crop/census unit combination. For an individual time-series, if the 95% confidence interval of the slope of the post-2000 segment does not overlap with the 95% confidence interval of the slope of the pre-2000 segment and the pre-2000 slope is greater than zero, and the post-2000 slope is lower than the pre-2000 slope, it is considered a case showing significant yield decrease. For a subset of cases where the 95% confidence interval of post-2000 slope overlaps with zero, it is considered a case showing yield stagnation.

Table S7 Likelihood of stagnation subsequent to the year 2000 based on analysis of previous 15 years of trends in yield gap and actual yield trend (See Methods)

	**All census units**	**Y2000> 0.9AY2000**	**Census units with 20-year convergent yield gaps**	**Census units with 50-year convergent yield gaps**	**Census units with slope of actual yield in top quartile**	**Census units with slope of actual yield in top quartile AND yield gaps converging within 20yrs**	**Number of units with sig 15 year decrease in yield gap**	**Number of census units with sig yield decrease**	**Number of census units with sig stagnation**
	Percentage of census units where bi-linear fit of yield timeseries shows significant decrease from a positive growth rate to a growth rate whose lower bound of confidence interval is less than zero	*Percentage of census units with significant stagnation AND where value of linear fit of actual yield over interval 1986-2000 evaluated at year 2000 is greater than 90% of value of attainable yield in year 2000*	*Percentage of census units with significant stagnation AND where linear fit to yield gaps over interval 1986-2000 has significantly negative slope and median yield gap closure time before 2020*	*Percentage of census units with significant stagnation AND where linear fit to yield gaps over interval 1986-2000 has significantly negative slope and median yield gap closure time before 2050*	*Percentage of census units in the top quartile of slope values with significant stagnation*	*Percentage of census units in the top quartile of slope values AND median yield gap closure less than 20 yrs with significant stagnation*			
barley	34.6%	57.4%	68.0%	68.0%	59.5%	68.3%	97	1341	1339
cassava	14.2%	16.4%	42.4%	36.8%	24.0%	40.0%	76	587	587
maize	17.0%	21.4%	42.5%	43.1%	19.7%	46.6%	663	1956	1940
oilpalm	63.8%	55.6%	NA	NA	72.6%	NA	0	1054	1054
rapeseed	16.0%	30.7%	45.9%	40.5%	26.8%	47.5%	84	464	464
rice	25.5%	52.7%	54.9%	50.2%	41.1%	61.3%	725	1668	1604
sorghum	29.8%	50.9%	86.6%	86.9%	57.2%	92.6%	183	1267	1267
soybean	15.6%	28.8%	58.5%	53.5%	26.3%	61.8%	173	796	786
sugarcane	32.3%	29.9%	57.6%	58.9%	52.0%	60.2%	129	1021	1019
wheat	19.1%	45.6%	56.0%	56.0%	32.9%	58.7%	75	1425	1316

Sensitivity to confidence intervals

We carried out the analysis of yield stagnation likelihood with a relaxed confidence interval criterion to assure that the conclusion that yield gap closure correlates with future yield stagnation was not due to a small number of census units identified as undergoing yield stagnation.

Table S8 Relaxed-Confidence Interval analysis of likelihood of stagnation subsequent to year 2000 based on analysis of previous 15 years of trends in yield gap and actual yield trend (See Methods). Here, significance for all trends is based on 75% confidence intervals

	**All census units**	**Y2000> 0.9AY2000**	**Census units with 20-year convergent yield gaps**	**Census units with 50-year convergent yield gaps**	**Census units with slope of actual yield in top quartile**	**Census units with slope of actual yield in top quartile AND yield gaps converging within 20yrs**	**Number of units with sig 15 year decrease in yield gap**	**Number of census units with sig yield decrease**	**Number of census units with sig stagnation**
	Percentage of census units where bi-linear fit of yield timeseries shows significant decrease from a positive growth rate to a growth rate whose lower bound of confidence interval is less than zero (75% Confidence intervals)	*Percentage of census units with significant stagnation AND where value of linear fit of actual yield over interval 1986-2000 evaluated at year 2000 is greater than 90% of value of attainable yield in year 2000 (75% Confidence intervals)*	*Percentage of census units with significant stagnation AND where linear fit to yield gaps over interval 1986-2000 has significantly negative slope and median yield gap closure time before 2020 (75% Confidence intervals)*	*Percentage of census units with significant stagnation AND where linear fit to yield gaps over interval 1986-2000 has significantly negative slope and median yield gap closure time before 2050 (75% Confidence intervals)*	*Percentage of census units in the top quartile of slope values with significant stagnation (75% Confidence intervals)*	*Percentage of census units in the top quartile of slope values AND median yield gap closure less than 20 yrs with significant stagnation (75% Confidence intervals)*			
barley	48.3%	70.2%	83.2%	83.2%	69.5%	84.3%	405	1933	1868
cassava	20.2%	23.7%	33.0%	30.3%	36.8%	46.2%	579	856	835

maize	24.3%	30.1%	48.8%	49.9%	26.8%	51.7%	1044	2987	2771
oilpalm	72.0%	71.2%	100.0%	100.0%	77.0%	100.0%	2	1213	1189
rapeseed	27.3%	51.0%	55.2%	37.6%	36.9%	68.9%	430	821	792
rice	33.9%	62.7%	61.2%	53.1%	48.8%	68.5%	1542	2477	2129
sorghum	39.8%	61.1%	80.8%	80.7%	67.8%	91.3%	643	1727	1693
soybean	24.6%	38.3%	73.2%	72.0%	38.6%	74.8%	296	1296	1241
sugarcane	44.5%	52.6%	67.9%	68.3%	72.2%	77.5%	609	1430	1405
wheat	28.9%	52.7%	49.6%	49.9%	42.5%	51.1%	521	2301	1987

Definition of Regions

The figure below shows the definitions of regions used in the analysis.

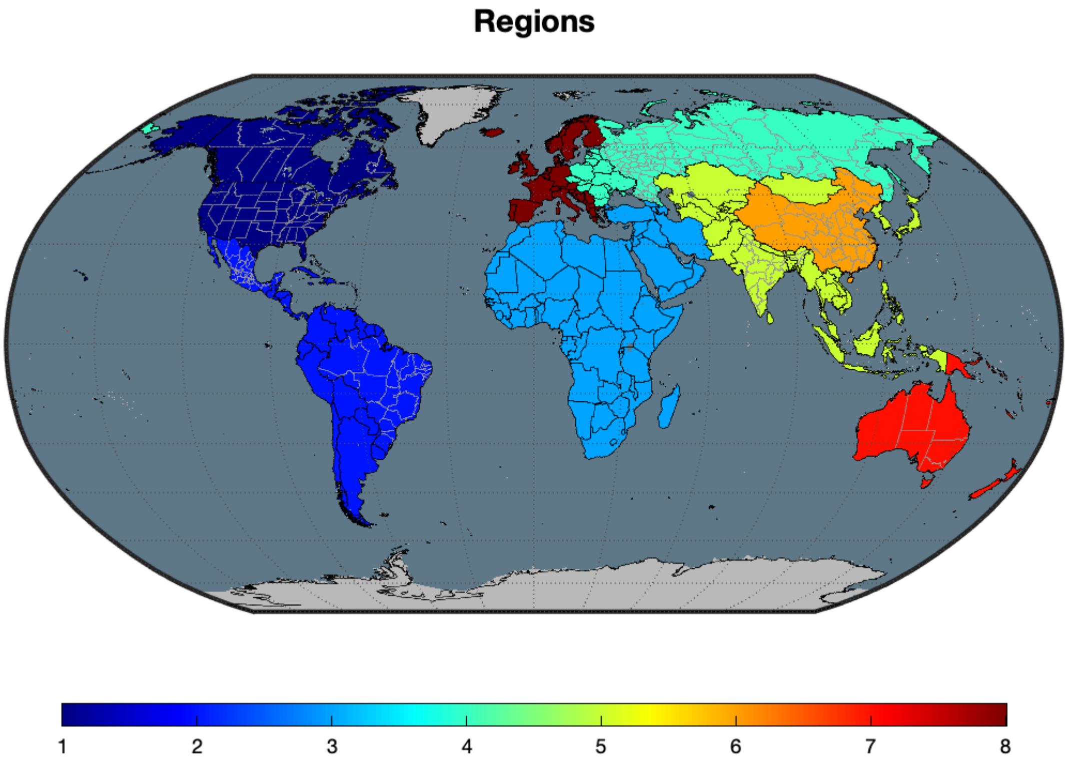

Figure S8 Representation of geographical regions. 1. North America; 2. Latin America; 3. Africa + Mideast; 4. Eastern Europe + Former Soviet Union (FSU);5. Asia (not including China); 6. China; 7. Oceania; 8. Western Europe

Dataset Selection

We carried out substantial portions of the analysis with three different datasets: A Climatology from WorldClim V2.1(Fick and Hijmans 2017), a climatology from CRU V4.05(Harris et al. 2020), and an annual climate from CRU V4.05(Harris et al. 2020). The WorldClim climatology led to the lowest temporal cross-validation error, suggesting it most appropriate for a study focused on interpretation of time trends. We tested the robustness of the results with regard to dataset selection by reproducing the data of relative yield gap growth rate (Table 3) comparing results based on the WorldClim climatology (those in the manuscript) to results based on the CRU annual weather data. (See Figure below).

Table S9 Cross-validation errors normalized to error of model with smallest error.

Source of temp/precip data	**Worldclim climatology**	**CRU climatology**	**CRU annual**
**barley**	1.279	**1.000**	1.016
**cassava**	**1.000**	1.115	1.032
**maize**	**1.000**	1.529	1.040
**oilpalm**	**1.000**	1.265	1.020
**rapeseed**	1.051	1.055	**1.000**
**rice**	**1.000**	1.109	1.082
**sorghum**	1.091	1.765	**1.000**
**soybean**	**1.000**	1.047	1.166
**sugarcane**	**1.000**	1.090	1.066
**wheat**	**1.000**	1.074	1.015

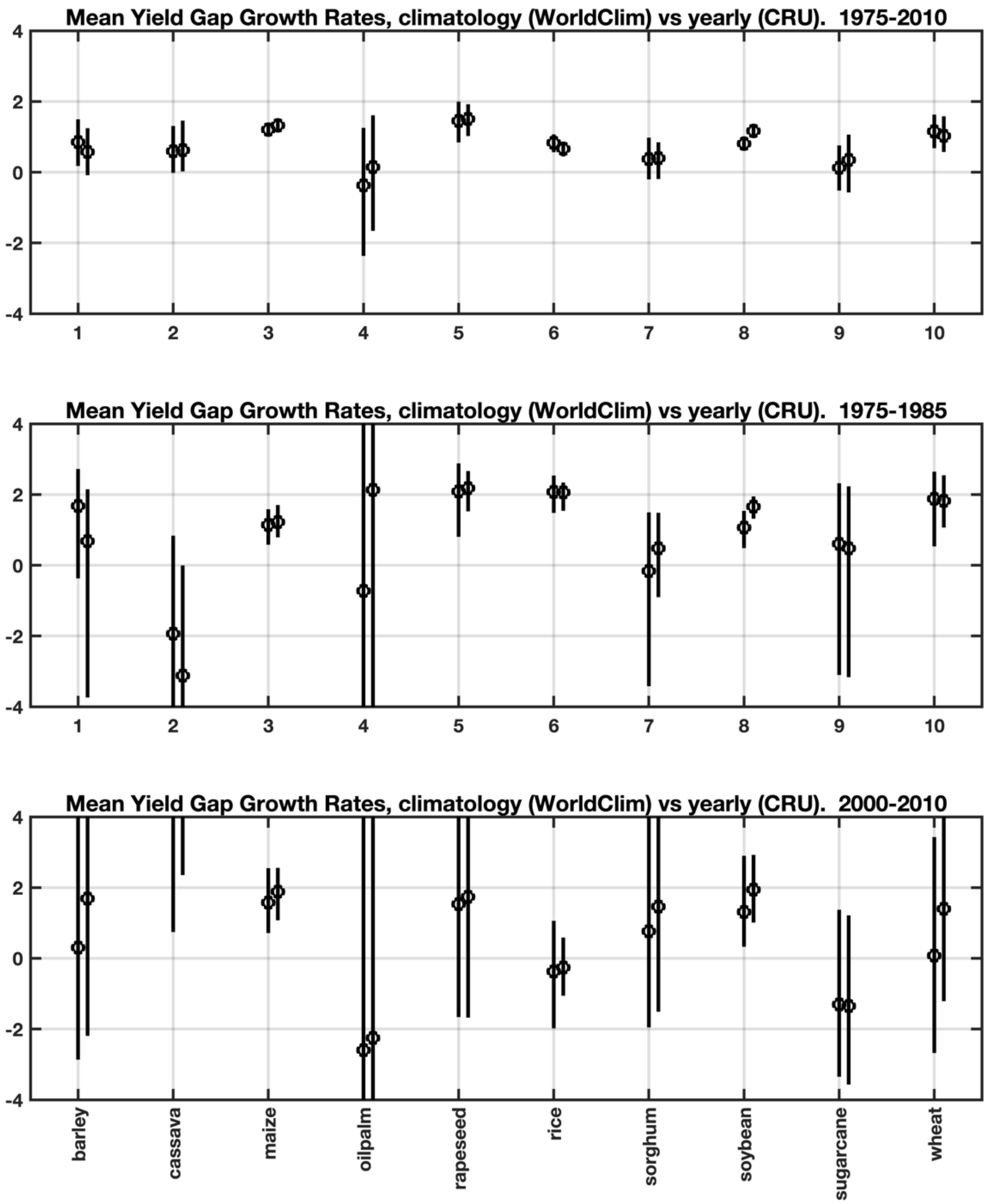

Figure S9. Mean yield gap growth rates and 95th percentile confidence intervals as calculated in the manuscript using the WorldClim climatology (left datapoints) and calculated using yearly weather data from CRU (right datapoints). Compare these results to Table 3 in the manuscript. Error bars for CRU analysis based on 100 realizations.

References

Fick, Stephen E., and Robert J. Hijmans. 2017. “WorldClim 2: New 1-Km Spatial Resolution Climate Surfaces for Global Land Areas.” *International Journal of Climatology* 37(12):4302–15.

Harris, I., Osborn, T. J., Jones, P. & Lister, D. Version 4 of the CRU TS monthly high-resolution gridded multivariate climate dataset. *Sci. Data* **7**, 1–18 (2020).

FAO: Fischer, G., Nachtergaele, F.O., van Velthuizen, H.T., Chiozza, F., Franceschini, G., Henry, M., Muchoney, D. and Tramberend, S. 2021. Global Agro-Ecological Zones v4 – Model documentation. Rome, FAO. https://doi.org/10.4060/cb4744enHarris, Ian, Timothy J. Osborn, Phil Jones, and David Lister. 2020. “Version 4 of the CRU TS Monthly High-Resolution Gridded Multivariate Climate Dataset.” *Scientific Data* 7(1):1–18. doi: 10.1038/s41597-020-0453-3.
