# Supplementary material for: Global spatially explicit yield gap time trends reveal regions at risk of future crop yield stagnation
Source: Nat Food. 2024 Jan 26;5(2):125–35. doi: 10.1038/s43016-023-00913-8 (PMC10896731; doi:10.1038/s43016-023-00913-8)
Supplement: Supplementary file 2 — Reporting Summary [file 43016_2023_913_MOESM2_ESM.pdf]

Corresponding author(s): James Gerber, jsgerber@umn.edu

Last updated by author(s): Oct 5, 2023

Reporting Summary

Nature Portfolio wishes to improve the reproducibility of the work that we publish. This form provides structure for consistency and transparency in reporting. For further information on Nature Portfolio policies, see our Editorial Policies and the Editorial Policy Checklist.

Statistics

For all statistical analyses, confirm that the following items are present in the figure legend, table legend, main text, or Methods section.

n/a	Confirmed	
☐	☒	The exact sample size (n) for each experimental group/condition, given as a discrete number and unit of measurement
☒	☐	A statement on whether measurements were taken from distinct samples or whether the same sample was measured repeatedly
☒	☐	The statistical test(s) used AND whether they are one- or two-sided<br>*Only common tests should be described solely by name; describe more complex techniques in the Methods section.*
☒	☐	A description of all covariates tested
☒	☐	A description of any assumptions or corrections, such as tests of normality and adjustment for multiple comparisons
☐	☒	A full description of the statistical parameters including central tendency (e.g. means) or other basic estimates (e.g. regression coefficient) AND variation (e.g. standard deviation) or associated estimates of uncertainty (e.g. confidence intervals)
☒	☐	For null hypothesis testing, the test statistic (e.g. F, t, r) with confidence intervals, effect sizes, degrees of freedom and P value noted<br>*Give P values as exact values whenever suitable.*
☒	☐	For Bayesian analysis, information on the choice of priors and Markov chain Monte Carlo settings
☒	☐	For hierarchical and complex designs, identification of the appropriate level for tests and full reporting of outcomes
☒	☐	Estimates of effect sizes (e.g. Cohen's d, Pearson's r), indicating how they were calculated

Our web collection on statistics for biologists contains articles on many of the points above.

Software and code

Policy information about availability of computer code

Data collection	No software was used for data collection.
Data analysis	All codes were developed in the Matlab and R programming languages. All codes associated with novel analysis are available at https://doi.org/10.5281/zenodo.10234041

For manuscripts utilizing custom algorithms or software that are central to the research but not yet described in published literature, software must be made available to editors and reviewers. We strongly encourage code deposition in a community repository (e.g. GitHub). See the Nature Portfolio guidelines for submitting code & software for further information.

Data

Policy information about availability of data

All manuscripts must include a data availability statement. This statement should provide the following information, where applicable:

- Accession codes, unique identifiers, or web links for publicly available datasets
- A description of any restrictions on data availability
- For clinical datasets or third party data, please ensure that the statement adheres to our policy

All weather, soil, and irrigation data used in this study are publicly available and sourced in references 66 and 68-73 of the methods, Crop yield and area data are derived from publicly available sources (agricultural census and survey reports as identified in Reference 58) as described and further referenced in the methods section. All data generated in the current study (Figures 1,2,4) as well as annual potential yield surfaces for ten crops over the period 1973-2012) can be

downloaded from https://doi.org/10.5281/zenodo.10234041. All data inputs to the study, as well as all results, are available upon request from the corresponding author. Authors commit to full and timely cooperation with any validation studies.

Research involving human participants, their data, or biological material

Policy information about studies with human participants or human data. See also policy information about sex, gender (identity/presentation), and sexual orientation and race, ethnicity and racism.

Reporting on sex and gender	N/A
Reporting on race, ethnicity, or other socially relevant groupings	N/A
Population characteristics	N/A
Recruitment	N/A
Ethics oversight	N/A

Note that full information on the approval of the study protocol must also be provided in the manuscript.

Field-specific reporting

Please select the one below that is the best fit for your research. If you are not sure, read the appropriate sections before making your selection.

☐ Life sciences ☐ Behavioural & social sciences ☒ Ecological, evolutionary & environmental sciences

For a reference copy of the document with all sections, see nature.com/documents/nr-reporting-summary-flat.pdf

Ecological, evolutionary & environmental sciences study design

All studies must disclose on these points even when the disclosure is negative.

Study description	Study of crop yield gaps based on compilations of publically available census data
Research sample	Census and survey data for yield and area for 10 crops over the period 1973-2012
Sampling strategy	No sampling was performed to obtain the dataset. Sub-sampling of the dataset was performed to assess confidence intervals.
Data collection	Crop data was collected by the second author.
Timing and spatial scale	Dataset is global, data are annual over period 1973-2012. Spatial scale depends on the number of administrative units within each country's agricultural census.
Data exclusions	No data were excluded.
Reproducibility	We have an entire ensemble of census data; there is no experiment to be repeated.
Randomization	Bootstrapping methods based on random sampling of the data were used to generate confidence intervals.
Blinding	n/a

Did the study involve field work? ☐ Yes ☒ No

Reporting for specific materials, systems and methods

We require information from authors about some types of materials, experimental systems and methods used in many studies. Here, indicate whether each material, system or method listed is relevant to your study. If you are not sure if a list item applies to your research, read the appropriate section before selecting a response.

Materials & experimental systems

n/a	Involved in the study
☒	☐ Antibodies
☒	☐ Eukaryotic cell lines
☒	☐ Palaeontology and archaeology
☒	☐ Animals and other organisms
☒	☐ Clinical data
☒	☐ Dual use research of concern
☒	☐ Plants

Methods

n/a	Involved in the study
☒	☐ ChIP-seq
☒	☐ Flow cytometry
☒	☐ MRI-based neuroimaging
